# Model for Musculoskeletal Injury Risk Factors Among US Army Basic Combat Trainees

**DOI:** 10.1001/jamanetworkopen.2025.13177

**Published:** 2025-06-02

**Authors:** Stephen A. Foulis, Susan P. Proctor, Barry A. Spiering, Leila A. Walker, Katelyn Guerriere Aaron, Colleen M. Castellani, Ian M. Hussian, Kristin J. Heaton, Mary L. Bouxsein, Erin Gaffney-Stomberg, Amy L. Fraley, Kristin L. Popp, Irene S. Davis, Jeffery S. Staab, Janet E. Staab, Jason L. Judkins, Shannon L. Merkle, Bradley M. Ritland, Ronald W. Matheny, Keith G. Hauret, Michelle Canham-Chervak, James P. McClung, Bruce H. Jones, Karl E. Friedl, Julie M. Hughes, Kathryn M. Taylor, and the ARIEM Reduction in Musculoskeletal Injury (ARMI) Study Team

**Affiliations:** 1 Military Performance Division, Army Research Institute of Environmental Medicine, Natick, Massachusetts; 2 Research Service, Veterans Affairs (VA) Boston Healthcare System, Boston, Massachusetts; 3 Endocrine Unit, Massachusetts General Hospital, Boston; 4 Center for Advanced Orthopedic Studies, Beth Israel Deaconess Medical Center, Boston, Massachusetts; 5 Department of Orthopedic Surgery, Harvard Medical School, Boston, Massachusetts; 6 Combat Feeding Division, Combat Capabilities Development Command–Soldier Center, Natick, Massachusetts; 7 Department of Exercise Science, University of South Carolina, Columbia; 8 School of Physical Therapy and Rehabilitation Sciences, University of South Florida, Tampa; 9 Military Operational Medicine Research Program, Fort Detrick, Maryland; 10 Defense Centers for Public Health-Aberdeen, Aberdeen, Maryland; 11 Military Nutrition Division, Army Research Institute of Environmental Medicine, Natick, Massachusetts; 12 Office of the Senior Scientist, Army Research Institute of Environmental Medicine, Natick, Massachusetts

## Abstract

**Importance:**

Musculoskeletal injuries (MSKIs) are pervasive problems in novice training environments. Evaluation of modifiable and nonmodifiable risk factors of MSKI risk prior to entry into these environments is largely understudied.

**Objective:**

To provide military leaders, civilian and military clinicians, and physical training instructors with an MSKI risk model for identifying low-, moderate-, and high-risk profiles among individuals starting US Army Basic Combat Training (BCT) or a physical training program.

**Design, Setting, and Participants:**

In this prospective cohort study, data collection was conducted between August 5, 2017, and April 15, 2023, at 2 US Army BCT sites. The sample consisted of volunteer trainees between the ages of 17 and 41 years. They were followed up from the start of BCT. Data analyses were conducted from April to September 2024.

**Exposures:**

Data for the factors potentially associated with MSKI were collected during the first week of BCT and included blood draws, total body dual-energy x-ray absorptiometry, and muscle power test results; surveys of demographics, medical history, physical activity, psychological characteristics, and sleep patterns; and physical fitness results.

**Main Outcomes and Measures:**

MSKIs identified using *International Statistical Classification of Diseases and Related Health Problems, Tenth Revision* (*ICD-10*) codes. Logistic regression–based models estimating the risk of MSKI were generated using 5-fold internal cross-validation for the total cohort, males, and females. Traffic light model examples of low (green), moderate (amber), and high (red) MSKI risk tiers were produced.

**Results:**

In this cohort study of 2988 Army trainees (median [IQR] age, 19.0 [19.0-22.0] years; 1880 males [62.9%]), 729 females (49.0%) and 758 males (51.0%) had an *ICD-10* code–identified MSKI, and 1067 (35.7%) had more than 1 *ICD-10* code–identified MSKI. Factors associated with increased MSKI risk in the total cohort and female- and male-specific MSKI risk models (with areas under the receiver operator characteristic curve of 0.701, 0.678, and 0.661, respectively) encompassed 7 variable categories: demographics; anthropometrics and body composition; nutritional status; medical and health history; history of sports and past or current physical activity or fitness; psychological factors (ie, pain, grit, and hardiness); and sleep parameters.

**Conclusions and Relevance:**

This cohort study presents a tiered approach to identifying persons at increased MSKI risk before the start of a physical training program. Applying a tiered quantification risk metric and incorporating multifactorial interventions from these findings may play a role in reduced MSKI risk.

## Key Points

**Question:**

What key factors present at the start of intense training programs, such as US Army Basic Combat Training, are associated with musculoskeletal injury (MSKI) risk during that period?

**Findings:**

In this cohort study of 2988 trainees, 27 modifiable and nonmodifiable factors were associated with MSKI risk during the 10-week basic combat training and follow-up period.

**Meaning:**

The results of this study support the use of a tiered quantification screening and multifactorial interventions prior to starting intense physical training programs to reduce the risk of MSKIs.

## Teaser

This cohort study identifies factors associated with the risk of sustaining musculoskeletal injuries during combat training or other physical training program.

## Introduction

Musculoskeletal injuries (MSKIs) are prevalent among athletes and individuals with physically demanding jobs, such as emergency responders and military personnel. For example, during US Army Basic Combat Training (BCT), MSKIs occur in 62% of women and 42% of men.^1,2^ BCT provides an ideal opportunity to evaluate risk factors for MSKI, with a controlled, 10-week physically demanding schedule.

Identifying risk factors is necessary to prevent MSKIs, with nonmodifiable factors useful for risk stratification. Such factors include older age,^1,3^ female sex,^3,4,5^ non-Hispanic White race,^6,7^ and prior injury.^1^ Modifiable factors, such as poor physical fitness,^4,8^ high and low body mass index (BMI),^4^ and tobacco use,^1,9^ provide targets for intervention. Several emerging factors warrant attention, such as nonsteroidal anti-inflammatory drugs,^10^ psychological characteristics,^11,12,13^ sleep,^14,15,16^ and reproductive function.^17^

Despite extensive research into MSKI risk factors and interventions in training environments, the rates of MSKI have remained consistently high during BCT.^18^ Current MSKI risk identification models developed for military^6,19,20^ and athlete^21^ populations have focused on risk factors present during military service or sport seasons. The key combination of factors present prior to physical training that impact MSKI risk during training is unknown.

To address this shortfall, we conducted a prospective cohort study involving trainees at the beginning of US Army BCT. Our aim was to provide military leaders, civilian and military clinicians, and physical training instructors with a traffic light MSKI risk model^20^ for identifying low-, moderate-, and high-risk profiles among individuals starting BCT or a physical training program.

## Methods

We analyzed data from the ARIEM Reduction in Musculoskeletal Injury (ARMI) Study,^22^ a prospective cohort study that received approval from the US Army Medical Research and Development Command Institutional Review Board. All participants were briefed on the methods and risks of participation and provided written informed consent^23^ to participate in the study and for investigators to obtain pertinent Army record information. We adhered to the policies for protection of human participants as prescribed in Army Regulation 70-25 and 45 CFR Part 46. We followed the Strengthening the Reporting of Observational Studies in Epidemiology (STROBE) reporting guideline.^24^

### Study Population

Trainees were enrolled in the ARMI Study between August 5, 2017, and April 15, 2023, at 2 BCT sites. These volunteer trainees were between the ages of 17 and 41 years. Females were oversampled to ensure appropriate sample sizes for sex-specific hypotheses and analyses. Power and sample size calculations were described in previous reports.^22^

### Data Collection

Data collection included standardized assessments of physical, mental, and lifestyle factors across the 10-week BCT cycle in BCT training cohorts. In these analyses, each participant was followed up for the 10 weeks during and the 6 weeks after BCT to track BCT-associated MSKIs using Army medical record systems data (the defined metric for BCT-associated MSKIs).

Methods were reported in detail elsewhere.^22^ Briefly, within the first 5 days of starting BCT, participants completed fasting blood draws; anthropometric measurements; body composition measurements via dual-energy x-ray absorptiometry (DXA; Prodigy, GE Healthcare); a vertical jump test to assess lower body strength or power (Vertec; Sports Imports); surveys ascertaining physical activity, health, and wellness histories; and validated measures of grit (12-item Grit Scale,^25^ including consistency, ambition, and perseverance subscales), hardiness (Dispositional Resilience Scale–II Military Version^26^), sleep characteristics (Pittsburgh Sleep Quality Index^27^ [PSQI]), and pain catastrophizing (Pain Catastrophizing Scale^28^ [PCS]). Race and ethnicity were self-reported by the participants and categorized according to Army guidance (eMethods in Supplement 1). Entry physical fitness scores from the Army Occupational Physical Assessment Test (OPAT)^29^ were collected from administrative records. Details of all data collection methods and variables are described in the eMethods and eTable 1 in Supplement 1.

The outcome of interest was MSKIs identified using *International Statistical Classification of Diseases and Related Health Problems, Tenth Revision* (*ICD-10*) codes from the Army medical encounter histories and based on the taxonomy developed by the Defense Centers for Public Health-Aberdeen.^30^ In BCT, all trainee sick-call visits to medical care are documented in medical encounter data. The injury metrics related to BCT included all injuries to the musculoskeletal system that met the taxonomy definition, including traumatic and cumulative microtraumatic injuries, for MSKIs occurring during BCT and in the 6 weeks after BCT as defined in the Army surveillance guidance.^31^ MSKI and OPAT data were obtained from the SPHERE (Soldier Performance, Health, and Readiness) research data repository, which aggregates Army record systems for the Army population for research purposes.

Seven variable groupings were considered for inclusion in the MSKI risk models. These categories were demographics, anthropometrics and body composition, nutritional status, medical and health history, history of sports and past or current physical activity or fitness, psychological factors (pain, grit, and hardiness), and sleep parameters.

### Statistical Analysis

Initial variable selection was based on prior evidence and consultation with MSKI subject matter experts, with a focus on metrics collected most efficiently in a large group setting. Highly correlated variables (*r* > 0.95) (Table 1; eTable 1 in Supplement 1) were excluded. Descriptives for all considered variables are provided in Table 2. Multiple imputation (5 imputations) was used to account for missing data.^32^ Details on missing data by variable are provided in eTable 1 in Supplement 1.

**Table 1.** Variables of Clinical Relevance and Scientific Evidence Considered and Included in Final Models

Variables considered and labels assigned in the models	Variables included		
	Total cohort model	Female-specific model	Male-specific model
**Demographics**			
Age, y	Yes	Yes	Yes
Sex	Yes	No	No
(0) Male			
(1) Female			
Race and ethnicity^a^	Yes	Yes	Yes
(1) African American or Black, non-Hispanic			
(2) Asian, non-Hispanic			
(3) Hispanic			
(4) Other, non-Hispanic^b^			
(5) White, non-Hispanic			
**Anthropometrics and body composition**			
Weight, kg	No	No	No
Height, cm	No	No	No
BMI	Yes	Yes	No
<18.5			
18.5-24.9			
25.0-29.9			
≥30.0			
DXA body fat, %	Yes	Yes	Yes
DXA lean mass, kg	No	No	No
DXA TBLH BMC, g	Yes	Yes	No
DXA TBLH BMD, g/cm^2^	Yes	No	Yes
**Nutritional status **			
Vitamin D (25-hydroxyvitamin D), ng/mL	Yes	No	Yes
Iron, μg/dL	Yes	No	No
Calcium, mg/dL	No	No	No
Ionized calcium, mmol/L	No	No	No
Total iron-binding capacity, μg/dL	No	Yes	Yes
Ferritin, ng/mL	Yes	No	Yes
**Medical and health history**			
Tobacco use	Yes	No	Yes
(1) Yes			
(0) No			
NSAID use	No	No	No
(1) Yes			
(0) No			
History of stress fracture	Yes	No	Yes
(1) Yes			
(0) No			
History of broken bone	Yes	Yes	Yes
(1) Yes			
(0) No			
History of concussion	Yes	No	No
(1) Yes			
(0) No			
**Females only**			
Age of first period	NA	No	NA
(1) <10 y			
(2) 10-12 y			
(3) 13-15 y			
(4) >15 y			
History of amenorrhea	NA	No	NA
(1) Yes			
(0) No			
History of contraceptive use	NA	No	NA
(1) Yes			
(0) No			
Current contraceptive use	NA	No	NA
(1) Yes			
(0) No			
**History of sports and past or current physical activity or fitness**			
Sport loading classification, before BCT	No	Yes	Yes
(1) Never played sports			
(2) Played nonmultidirectional sports			
(3) Played multidirectional sports			
Exercise frequency per wk in 2 mo before BCT	Yes	Yes	No
(1) 0-2 times/wk			
(2) 3-4 times/wk			
(3) ≥5 times/wk			
Exercise in y before BCT vs prior 2 mo before BCT	Yes	Yes	Yes
(1) Much less			
(2) Somewhat less			
(3) About the same			
(4) Somewhat more			
(5) Much more			
Physical activity level rating: Compared to others your same age and sex, how would you rate yourself on the level of physical activity prior to BCT?	Yes	Yes	Yes
(1) Much less active			
(2) Somewhat less active			
(3) About the same			
(4) Somewhat more active			
(5) Much more active			
Running: Over the last 2 mo prior to BCT, how many times per week did you run or jog?	Yes	Yes	Yes
(1) 0-2 times/wk			
(2) 3-4 times/wk			
(3) ≥5 times/wk			
Weight training: Over the last 2 mo prior to BCT, how often did you perform weight training exercises?	Yes	No	Yes
(1) 0-2 times/wk			
(2) 3-4 times/wk			
(3) ≥5 times/wk			
Vertical jump test average, cm	No	No	No
OPAT dead lift, kg	No	No	No
OPAT long jump distance, cm	Yes	No	No
OPAT power throw distance, cm	Yes	Yes	No
OPAT interval run, No. of levels completed	No	No	No
OPAT IR, total shuttles completed	No	No	No
**Psychological factors**			
Total Grit Scale summary score	Yes	No	Yes
Hardiness: positive summary score	No	No	No
Hardiness: negative summary score	Yes	Yes	No
Total PCS score	Yes	No	Yes
**Sleep parameters**			
PSQI global sleep score	Yes	Yes	Yes
PSQI pre-BCT sleep duration	Yes	No	Yes
(0) >7 h			
(1) 6-7 h			
(2) 5-6 h			
(3) <5 h			
PSQI pre-BCT sleep quality	Yes	Yes	No
(0) Very good			
(1) Fairly good			
(2) Fairly bad			
(3) Very bad			
PSQI pre-BCT sleep efficiency	No	No	No
(0) >84.5%			
(1) >74.5%-84.5%			
(2) 65%-74.5%			
(3) <65%			

Abbreviations: BCT, basic combat training; BMC, bone mineral content; BMD, bone mineral density; BMI, body mass index (calculated as weight in kilograms divided by height in meters squared); DXA, dual-energy x-ray absorptiometry; NA, not applicable; NSAID, nonsteroidal anti-inflammatory drug; OPAT, Occupational Physical Assessment Test; PCS, Pain Catastrophizing Scale; PSQI, Pittsburgh Sleep Quality Index; TBLH, total body less head.

SI conversion factor: To convert calcium to millimole per liter, multiply by 0.25; ferritin to microgram per liter, multiply by 1.0; iron and total iron-binding capacity to micromole per liter, multiply by 0.179; vitamin D to nanomole per liter, multiply by 2.496.

^a^ Race and ethnicity were self-identified and categorized according to Army guidance.

^b^ Other included participants who identified as multiracial, Native American or Alaska Native, Indian or from Indian subcontinent, or Native Hawaiian or Pacific Islander.

**Table 2.** Descriptions of Variables in Total Cohort MSKI Risk Model

Variable and description (coding)	No. (%)			Univariate results	
	Total population (n = 2988)	With MSKI (n = 1487)	Without MSKI (n = 1501)	OR (95% CI)	*P* value
**Demographics**					
Age, median (IQR), y	19.0 (18.0-22.0)	19.0 (18.0-22.0)	19.0 (18.0-22.0)	1.02 (1.00-1.04)	.02
Sex					
Male	1880 (62.9)	758 (51.0)	1122 (74.8)	1 [Reference]	NA
Female	1108 (37.1)	729 (49.0)	379 (25.3)	2.85 (2.44-3.32)	<.001
Race and ethnicity^a^					
African American or Black, non-Hispanic	620 (20.9)	329 (22.3)	291 (19.5)	1.37 (1.12-1.66)	.002
Asian, non-Hispanic	134 (4.5)	69 (4.7)	65 (4.4)	1.29 (0.90-1.84)	.16
Hispanic	705 (23.8)	369 (25.1)	336 (22.6)	1.32 (1.09-1.59)	.004
White, non-Hispanic	1240 (41.9)	564 (38.3)	676 (45.4)	1 [Reference]	NA
Other, non-Hispanic^b^	264 (8.9)	142 (9.6)	122 (8.2)	1.40 (1.07-1.82)	.01
**Anthropometric and body composition**					
BMI					
<18.5	47 (1.6)	29 (2.0)	18 (1.2)	1.51 (0.83-2.75)	.17
18.5-24.9	1515 (51.2)	775 (52.6)	740 (49.9)	1 [Reference]	NA
25.0-29.9	1176 (39.8)	573 (38.9)	603 (40.7)	0.91 (0.78-1.06)	.23
≥30.0	219 (7.4)	97 (6.6)	122 (8.2)	0.78 (0.59-1.03)	.08
DXA body fat mean (SD), %	26.57 (7.63)	28.26 (7.59)	24.84 (7.28)	1.06 (1.05-1.08)	<.001
DXA-BMC, mean (SD), g/cm^2^	2763.50 (511.76)	2643.86 (505.25)	2886.06 (489.04)	0.99 (0.99-0.99)	<.001
DXA-BMD, mean (SD), g/cm^2^	1.24 (0.14)	1.21 (0.13)	1.26 (0.13)	0.97 (0.97-0.98)	<.001
**Nutritional status**					
Vitamin D (25-hydroxyvitamin D), mean (SD), ng/mL	24.34 (8.96)	23.67 (8.75)	24.99 (9.11)	0.99 (0.98-0.99)	<.001
Iron, mean (SD), μg/dL	71.84 (32.12)	68.50 (32.15)	75.13 (31.76)	0.99 (0.99-0.99)	<.001
Ferritin, median (IQR), ng/mL	79.40 (37.55-133.00)	64.50 (28.45-119.00)	90.55 (51.00-144.50)	0.99 (0.99-0.99)	<.001
**Medical and health history**					
Tobacco use					
Yes	698 (23.6)	357 (24.2)	341 (23.0)	1.07 (0.90-1.27)	.44
No	2265 (76.4)	1120 (75.8)	1145 (77.1)	1 [Reference]	NA
History of stress fracture					
Yes	97 (3.3)	56 (3.8)	41 (2.8)	1.35 (0.90-2.03)	.15
No	2828 (96.7)	1405 (96.2)	1423 (97.2)	1 [Reference]	NA
History of broken bone					
Yes	592 (20.2)	311 (21.2)	281 (19.1)	1.14 (0.95-1.36)	.17
No	2341 (79.8)	1153 (78.8)	1188 (80.9)	1 [Reference]	NA
History of concussion					
Yes	319 (10.9)	157 (10.7)	162 (11.1)	0.96 (0.77-1.21)	.75
No	2608 (89.1)	1306 (89.3)	1302 (88.9)	1 [Reference]	NA
**History of sports and past or current physical activity or fitness**					
Exercise frequency per week in 2 mo before BCT					
0-2 times/wk	1068 (35.7)	622 (41.8)	446 (29.7)	1.88 (1.57-2.26)	<.001
3-4 times/wk	1066 (35.7)	500 (33.6)	566 (37.7)	1.19 (0.99-1.44)	.06
≥5 times/wk	811 (27.1)	345 (11.6)	466 (31.1)	1 [Reference]	NA
Exercise in the y before BCT vs 2 mo before BCT					
Much less	536 (18.3)	294 (20.1)	242 (16.4)	1.49 (1.20-1.84)	<.001
Somewhat less	575 (19.6)	292 (20.0)	283 (19.2)	1.26 (1.02-1.56)	.03
About the same	914 (31.1)	411 (28.2)	503 (34.3)	1 [Reference]	NA
Somewhat more	521 (17.8)	258 (17.7)	263 (17.7)	1.21 (0.97-1.50)	.09
Much more	389 (13.3)	207 (14.2)	182 (12.4)	1.40 (1.10-1.77)	.006
Physical activity level: Compared to others your same age and sex, how would you rate yourself on the level of physical activity prior to BCT?					
Much less active	345 (11.7)	209 (14.3)	136 (9.2)	1.64 (1.26-2.14)	<.001
Somewhat less active	756 (25.7)	440 (30.0)	316 (21.4)	1.50 (1.22-1.85)	.001
About the same	683 (23.2)	329 (22.4)	354 (23.9)	1 [Reference]	NA
Somewhat more active	805 (27.3)	336 (22.9)	469 (31.7)	0.77 (0.63-0.94)	.01
Much more active	356 (12.1)	152 (10.4)	204 (13.8)	0.80 (0.62-1.04)	.10
Running: Over the last 2 mo prior to BCT, how many times per week did you run or jog?					
0-2 times/wk	1640 (54.9)	893 (60.1)	747 (50.2)	1.57 (1.34-1.84)	<.001
3-4 times/wk	977 (32.7)	422 (28.4)	555 (37.3)	1 [Reference]	NA
≥5 times/wk	313 (10.5)	144 (9.7)	169 (11.4)	1.12 (0.87-1.45)	.39
Weight training: Over the last 2 mo prior to BCT, how often did you perform weight training exercises?					
0-2 times/wk	1826 (61.1)	980 (65.9)	846 (56.4)	1.47 (1.23-1.74)	<.001
3-4 times/wk	713 (23.9)	317 (21.3)	396 (26.4)	1 [Reference]	NA
≥5 times/wk	394 (13.2)	165 (11.1)	229 (15.3)	0.91 (0.71-1.74)	.44
OPAT long jump distance, mean (SD), cm	178.73 (32.78)	177.92 (32.33)	179.55 (33.22)	1.00 (0.99-1.00)	.55
OPAT power throw distance, mean (SD), cm	504.06 (102.20)	504.99 (103.54)	503.12 (100.87)	1.01 (0.99-1.02)	.29
**Psychological factors**					
Total Grit Scale summary score, mean (SD)	42.34 (6.52)	42.53 (6.51)	42.16 (6.52)	1.01 (0.99-1.02)	.29
Hardiness: negative summary score, mean (SD)	29.22 (6.85)	29.38 (7.00)	29.07 (6.70)	1.01 (0.99-1.02)	.16
Total PCS, median (IQR)	8.00 (2.00-16.00)	9.00 (2.00-17.00)	8.00 (2.00-16.00)	1.01 (1.00-1.02)	.04
**Sleep parameters**					
PSQI global sleep score, median (IQR)	4.00 (3.00-6.00)	5.00 (3.00-7.00)	4.00 (3.00-6.00)	1.07 (1.04-1.09)	<.001
PSQI sleep duration, pre-BCT					
>7 h	1626 (56.5)	812 (56.5)	814 (56.5)	1 [Reference]	NA
6-7 h	901 (31.3)	432 (30.0)	469 (32.6)	0.93 (0.79-1.09)	.36
5-6 h	212 (7.4)	111 (7.7)	101 (7.0)	1.13 (0.85-1.50)	.39
<5 h	140 (4.9)	83 (5.8)	57 (4.0)	1.50 (1.07-2.12)	.02
PSQI sleep quality, pre-BCT					
Very good	716 (25.0)	350 (24.5)	366 (25.5)	1 [Reference]	NA
Fairly good	1621 (56.7)	804 (56.3)	817 (57.0)	1.04 (0.88-1.24)	.64
Fairly bad	432 (15.1)	215 (15.1)	217 (15.1)	1.04 (0.82-1.31)	.76
Very bad	92 (3.2)	59 (4.1)	33 (2.3)	1.88 (1.20-2.93)	.006

Abbreviations: BCT, basic combat training; BMC, bone mineral content; BMD, bone mineral density; BMI, body mass index (calculated as weight in kilograms divided by height in meters squared); DXA, dual-energy x-ray absorptiometry; MSKI, musculoskeletal injury; NA, not applicable; OPAT, Occupational Physical Assessment Test; PCS, Pain Catastrophizing Scale; PSQI, Pittsburgh Sleep Quality Index.

SI conversion factor: To convert ferritin to microgram per liter, multiply by 1.0; iron to micromole per liter, multiply by 0.179; vitamin D to nanomole per liter, multiply by 2.496.

^a^ Race and ethnicity were self-identified and categorized according to Army guidance.

^b^ Other included participants who identified as multiracial, Native American or Alaska Native, Indian or from Indian subcontinent, or Native Hawaiian or Pacific Islander.

Factors associated with MSKI were assessed within the total cohort and separately stratified by sex. The final models generated probability of injury (Pr(MSKI)) metrics, ranging from 0 to 1, with higher values reflecting greater risk of MSKI. MSKI risk models are interpreted as the summation of each of its risk factors. While some variables may not reach statistical significance individually, they may still contribute to the overall risk of MSKI. To compute the probability of injury, the following formula was followed: 1/1 + *e*^(β0 + β1^*^X^*^1 + β2^ *^X^*^2 …)^, where (β_0_+β_1_*X*_1_+β_2_ *X*_2_ …) represents the sum of the model values. Logistic regression models output the logit(Pr(MSKI)).

Data were stacked and a weight was applied for variable selection to account for imputed data.^33^ To reduce the potential for overfitting, final models were determined using an elastic net variable selection^34^ as a regularization technique, which accounts for correlated or clustered data.

Internal cross-validation was performed using k-fold cross-validation with 5-folds, randomly splitting the data into 5 equal datasets.^35^ The best-fit model was built in 4 of the datasets and then tested in the 1 holdout dataset and repeated 5 times. After variables were determined across all folds, the final model was estimated for the entire dataset.

As the goal of the study was to provide clinicians, instructors, and military leaders with guidance on identifying individuals most at risk for injury, a traffic light paradigm using the Youden index^36^ was applied to each model-specific area under the receiver operating characteristic curve (AUROC) to derive the cut point indicators of MSKI probability that produced maximal sensitivity and specificity. This cut point was used as the threshold, where moderate MSKI risk (amber) was defined as above the Youden index cut point, and low MSKI risk (green) was defined as below the cut point. A high MSKI risk (red) was computed as halfway between the Youden index cut point and a probability of 1. For both the moderate and high-risk cut points, the sensitivity, specificity, positive predictive value (PPV), and negative predictive value (NPV) were calculated for model performance, along with the observed and predicted number of persons injured within those risk tiers. Several dynamic multivariate profile examples were included to demonstrate how the traffic light heuristic could be computed.

Two-sided *P* < .05 indicated statistical significance. All analyses were conducted from April to September 2024 using SAS version 9.4 (SAS Institute Inc).

## Results

Of the 3886 trainees enrolled in the ARMI study, 2988 were included in the analyses. Reasons for exclusion were related to lack of data on MSKI outcome or missing survey data (Figure). In this analytic cohort (median [IQR] age, 19.0 [19.0-22.0] years; 1108 females [37.1%] and 1880 males [62.9%]) (Table 2), 729 females (49.0%) and 758 males (51.0%) had an *ICD-10* code–identified MSKI, and 1067 (35.7%) had more than 1 *ICD-10* code–identified MSKI. Injuries identified by pain-related symptom diagnostic codes were the most prevalent among persons with MSKIs (n = 1487) (Figure). After excluding pain symptom diagnoses, the top 3 MSKI-specific diagnoses were ankle or foot sprain (n = 77), tibia or fibula stress fracture (n = 49), and pelvic stress fracture (n = 41).

**Figure.**
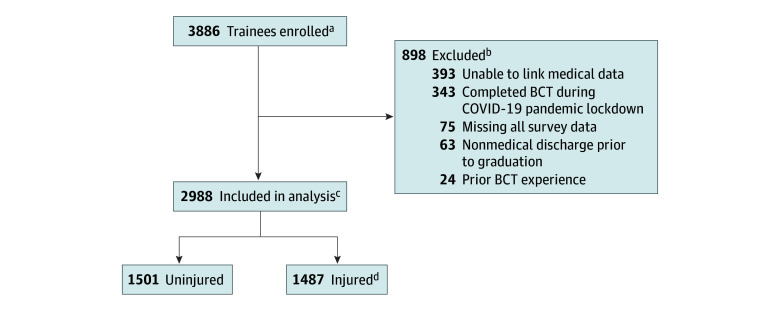
Diagram of Analytic Cohort BCT indicates basic combat training. ^a^Enrolled trainees comprised 2507 males (64.5%) and 1379 females (35.5%) with a mean (SD) age of 21.01 (3.89) years. ^b^Participants were excluded during the COVID-19 pandemic lockdown because their training regimens were not comparable with those of the others. ^c^Final sample included 1879 males (62.9%) male and 1109 females (37.1%) with a mean (SD) age of 20.89 (3.84) years. ^d^A total of 3070 injuries were recorded among 1487 persons injured. The 3 most prevalent injury types (in terms of percent of trainees experiencing the injury) were upper extremity (n = 314), lower extremity (n = 1334), and soft tissue (n = 1422).

### Final MSKI Risk Models

There were 45 variables considered for evaluation as factors potentially associated with MSKI (Table 1). Twenty-seven variables, from all 7 variable categories, made up the total cohort MSKI risk model (Table 1 and Table 2). A summary of the total cohort risk model variables and metrics is provided in the eResults in Supplement 1. Univariate results for variables included in the total cohort MSKI risk model are presented in Table 2. Eighteen out of the 27 variables selected into the final model were associated with MSKI when examined univariately.

The total cohort MSKI risk model demonstrated higher risk of MSKI as age increased in females compared with males (odds ratio [OR], 1.06 [95% CI, 1.03-1.08] vs 1.29 [95% CI, 0.94-1.77]) and among various race and ethnic categories compared with non-Hispanic White individuals. With respect to body composition, persons considered underweight compared with healthy weight, as measured by BMI (OR, 1.59; 95% CI, 0.84-3.03), were at higher risk of MSKI. Persons with higher body fat percentage (OR, 1.05; 95% CI, 1.03-1.07) or lower total body bone mineral content (OR, 1.00; 95% CI, 1.00-1.00) had increased risk of MSKI, and those with a higher bone mineral density (OR, 0.20; 95% CI, 0.05-0.80) had lower risk. Higher levels of serum vitamin D (OR, 1.00; 95% CI, 0.99-1.01), iron (OR, 1.00; 95% CI, 1.00-1.00), or ferritin (OR, 1.00; 95% CI, 1.00-1.00) were associated with lower MSKI risk. Trainees reporting tobacco use (OR, 1.22; 95% CI, 1.01-1.47) or a history of a broken bone (OR, 1.36; 95% CI, 1.11-1.67), stress fracture (OR, 1.30; 95% CI, 0.83-2.03), or concussion prior to BCT were at higher risk of MSKI. A lower frequency of exercise per week in the 2 months prior to BCT compared with more than 5 times per week prior to BCT was associated with an increased risk of MSKI. Those reporting more exercise in the year before BCT compared with 2 months before were generally at higher risk of MSKI. Physical activity, reported as higher or lower than one’s peers, or a higher and lower frequency of running or weightlifting compared with 3 to 4 times per week in the 2 months prior to BCT presented an increased risk in a U-shaped relationship. Lower OPAT long jump (OR, 1.00; 95% CI, 0.99-1.00) and longer OPT power throw (OR, 1.00; 95% CI, 1.00-1.00) distances were associated with increased risk. In terms of psychological factors, higher scores on grit (OR, 1.02; 95% CI, 1.00-1.03), negative hardiness (OR, 1.01; 95% CI, 0.99-1.02), and pain catastrophizing (OR, 1.00; 95% CI, 0.99-1.01) scales were associated with higher risk. Poorer sleep, as measured by the PSQI global score (OR, 1.08; 95% CI, 1.03-1.13), was associated with increased risk of MSKI. Similarly, increased risk was observed among those who reported less than 5 hours of sleep per night compared with over 7 hours per night or those reporting very bad sleep quality compared with very good. The total cohort MSKI risk model metrics, including ORs (95% CIs) for each variable, are provided in eTable 2 in Supplement 1.

Compared with the total cohort MSKI risk model, there were few differences in the variables selected for the male- vs female-specific models (Table 1). In the female-specific model, the additional variables of higher total iron-binding capacity and sports load class (playing a unidirectional or nonimpact sport vs multidirectional sport) were associated with increased MSKI risk. Bone mineral density, iron, ferritin, vitamin D, prior stress fracture, history of concussion, tobacco use, frequency of weight training prior to BCT, vertical jump height, OPAT long jump distance, grit, pain catastrophizing, and ratings of sleep duration did not meet model inclusion criteria. In the male-specific model, additional risk factors included lower total iron-binding capacity and load class (both playing a unidirectional or nonimpact sport or no sports vs multidirectional sport). However, BMI, bone mineral content, iron, exercise frequency in 2 months prior to BCT, vertical jump height, OPAT long jump, OPAT power throw, negative hardiness, and ratings of sleep quality did not meet model inclusion criteria. Details are summarized in eResults in Supplement 1; male and female model metrics are provided in eTables 3-5 in Supplement 1.

The total cohort MSKI risk model performed slightly better with an AUROC equal to 0.701 compared with 0.678 and 0.661 for the female- and male-specific models, respectively (Table 3). The moderate risk cut point established by Youden index (0.482) for the total cohort MSKI risk model produced a sensitivity of 0.660, specificity of 0.647, PPV of 0.649, and NPV of 0.658. Applying the probability cut point that identifies the high MSKI risk population (0.741), the model succeeded at increasing the number of true injuries captured (PPV = 84%) and reducing the number of false injuries (specificity), at the cost of reducing the sensitivity or classifying more people with true injuries as uninjured. However, some of these missed injured people were captured by the moderate risk threshold.

**Table 3.** Model Criteria Metrics for Traffic Light Risk Tiers

Cohort and tier^a^	Probability cut point	Observed No. of injured people	Predicted No. of injured people	AUROC	Sensitivity	Specificity	PPV	NPV
Total cohort								
High risk	0.741	253	301	0.701	0.170	0.968	0.841	0.541
Moderate risk	0.482	729	1212	0.701	0.660	0.647	0.649	0.658
Females								
High risk	0.821	121	139	0.678	0.166	0.951	0.868	0.372
Moderate risk	0.641	363	506	0.678	0.664	0.545	0.750	0.471
Males								
High risk	0.704	37	47	0.661	0.049	0.991	0.787	0.603
Moderate risk	0.407	417	713	0.661	0.559	0.639	0.529	0.703

Abbreviations: AUROC, area under the receiver operating characteristic curve; NPV, negative predictive value; PPV, positive predictive value.

^a^ Traffic light tiers were red for high risk, amber for moderate risk, and green for low risk.

Examples of high-, moderate-, and low-risk profiles for MSKI are specified in the Box. A computation spreadsheet is presented in eAppendix in Supplement 2.

Box. **Example Scenarios of Traffic Light Musculoskeletal Injury Risk Tiers^a^**

**Example 1: Green (low risk)**

The probability of injury is approximately 46.34% (95% CI, 44.55%-48.12%) for a 20-year-old (male median) White (reference) male with no history of a broken bone or stress fracture before BCT (reference), overweight BMI (between 25-29.9), body fat of 16.44% (1 SD below male mean), DXA-BMC of 3455.8 g (1 SD above male mean), DXA-BMD of 1.49 g/cm^2^ (1.5 SD above male mean), vitamin D level of 33.34 ng/mL (1.5 SD above male mean), iron level of 109.54 μg/dL (1 SD above male mean), ferritin level of 165.0 ng/nL (male 75th percentile), no prior tobacco use (reference), no history of concussion (reference), reported exercising 5 to 7 times per week in 2 months (reference) prior to BCT, reported exercising somewhat more in the year prior to vs 2 months prior to BCT, reported physical activity rating in the year prior to BCT as somewhat more active compared with peers, reported running 3 to 4 times per week (reference) in 2 months prior to BCT, reported lifting weights 3 to 4 times per week (reference) in 2 months prior to BCT, OPAT long jump distance of 230.7 cm (1.5 SD above male mean), OPAT power throw distance of 509.6 cm (male mean), total Grit Scale summary score of 42.06 (male mean), negative hardiness score of 29.41 m (male mean), total PCS of 8.00 (male median), PSQI score of 4.00 (male median), reported sleep duration as 6 to 7 hours per night on average vs over 7 hours per night (reference), and reported sleep quality as very good vs very bad (reference).

**Example 2: Amber (moderate risk)**

The probability of injury is approximately 65.09% (95% CI, 63.38%-66.80%) for a 20-year-old (male median) White (reference) male with no history of a broken bone or stress fracture before BCT (reference), healthy BMI (between 18.5-24.9 [reference]), body fat of 23.11% (male mean), DXA-BMC of 3016.8 g (male mean), DXA-BMD of 1.28 g/cm^2^ (male mean), vitamin D level of 24.48 ng/mL (male mean), iron level of 109.54 μg/dL (1 SD above male mean), ferritin level of 165.0 ng/nL (male 75th percentile), no prior tobacco use (reference), no history of concussion (reference), reported exercising 5 to 7 times per week in 2 months (reference) prior to BCT, reported exercising somewhat more in the year prior vs 2 months prior to BCT, reported physical activity rating in the year prior to BCT as about the same (reference) compared to peers, reported running 3 to 4 times per week (reference) in 2 months prior to BCT, reported lifting weights 3 to 4 times per week (reference) in 2 months prior to BCT, OPAT long jump distance of 214.10 cm (1 SD above male mean), OPAT power throw distance of 509.6 cm (male mean), total Grit Scale summary score of 42.06 (male mean), negative hardiness score of 29.41 (male mean), total PCS of 8.00 (male median), PSQI score of 4.00 (male median), reported sleep duration as over 7 hours per night (reference), and reported sleep quality as very good (reference).

**Example 3: Red (high risk)**

The probability of injury is approximately 96.39% (95% CI, 95.72%-97.06%) for a 20-year-old (male median) White (reference) male with a history of a broken bone before BCT but not a stress fracture (reference), obese BMI (≥30), body fat of 29.78% (1 SD above male mean), DXA-BMC of 2579.36 g (1 SD below male mean), DXA-BMD of 1.4 g/cm^2^ (1 SD below male mean), vitamin D level of 15.62 ng/mL (1 SD below male mean), iron level of 48.72 μg/dL (1 SD below male mean), mean ferritin level of 77.1 ng/nL (male 25th percentile), no prior tobacco use (reference), no history of concussion (reference), reported exercising 5 to 7 times per week in 2 months (reference) prior to BCT, reported exercising about the same in the year prior vs 2 months prior to BCT, reported physical activity rating in the year prior to BCT as about the same (reference) compared with peers, reported running 0 to 2 times per week in 2 months prior to BCT, reported lifting weights 0 to 2 times per week (reference) in 2 months prior to BCT, OPAT long jump distance of 147.35 cm (1 SD below male mean), OPAT power throw distance of 509.6 cm (male mean), total Grit Scale summary score of 42.06 (male mean), negative hardiness score of 29.41 (male mean), total PCS of 8.00 (male median), PSQI score of 15.00 (male 75th percentile), reported sleep duration as less than 5 hours per night on average vs over 7 hours (reference), and reported sleep quality as very bad vs very good (reference).

Abbreviations: BCT, basic combat training; BMC, bone mineral content; BMD, bone mineral density; BMI, body mass index (calculated as weight in kilograms divided by height in meters squared); DXA, dual-energy x-ray absorptiometry; OPAT, Occupational Physical Assessment Test; PCS, Pain Catastrophizing Scale; PSQI, Pittsburgh Sleep Quality Index.

^a^
The computation spreadsheet (locked) illustrating these examples is provided in eAppendix in Supplement 2.

## Discussion

This study provides a model for identifying MSKI risk in young Army trainees initiating BCT or a physical training program. Combinations of modifiable and nonmodifiable risk factors were found to increase or decrease MSKI risk and provide a foundation for risk stratification and risk reduction interventions.

The traffic light model provided tiered quantification of MSKI risk and could serve as the basis of a screening tool for Army and other medical personnel to use in identifying individuals at elevated risk at the beginning of physical activity programs. Military leaders or trainers could then assign stratified guidance to mitigate moderate-to-high MSKI risk, such as greater injury surveillance or enhanced training interventions before and during training. As depicted in the examples of the tiered risk models (Box; eAppendix in Supplement 2), it is the combination of risk factors that increases or decreases overall MSKI risk. For example, a person might demonstrate improvements in body composition through interventions, but continuing poor sleep patterns and/or extreme exercise training prior to BCT may negate the benefits to overall MSKI risk. Follow-up external validation to address generalizability of the risk models is under way.

### Key Risk Factors of MSKI

Similar to other retrospective^6,37^ and prospective^1,38^ studies in military populations, female sex, older age, race other than non-Hispanic White, and prior injury (eg, bone injury, stress fracture, and concussion) were key nonmodifiable risk factors for MSKI. The remaining risk factors were modifiable in nature and, taken together, provide opportunities for changing behaviors to minimize MSKI risk before initiating training programs.

#### Anthropometrics and Body Composition

Unlike most studies using medical records that limit anthropometric risk factors to height, weight, and BMI,^1,4^ this study included DXA measurements that allowed the investigation of the role of skeletal and soft tissue factors in MSKI. Consistent with other reports, we found that low BMD,^39^ high body fat percentage, and low BMI^4^ were associated with increased risk of various MSKIs.

#### Medical and Health History 

Smoking history has been shown to be a factor in MSKI^1,40^; however, in the present study, it was not selected for the female-specific model. The reason may be the changing patterns in the population; in our study, there were few females using tobacco-based products.

#### Nutritional Status 

Greater risk of bone stress injuries has been observed in females compared with males,^2^ and vitamin D level has been associated with stress fracture risk.^41^ However, we found that low vitamin D levels were risk factors for MSKI in the total cohort and male-specific models but not in the female-specific model. Specific sex differences in vitamin D level as a risk factor will be explored in planned future analyses of bone stress injury risk within this cohort. Lower ferritin levels were associated with increased lower limb overuse injury during training.^42^ The current study findings provide additional support for achieving adequate iron status prior to starting a training program.

#### History of Sports and Past or Current Physical Activity or Fitness

Moderate levels of aerobic activity (running) and resistance training (weightlifting) 3 to 4 times per week prior to BCT were associated with lower MSKI risk compared with both lower and higher exercise frequency rates. These nonlinear associations highlight the importance of not undertraining or overtraining prior to intense physical training programs such as BCT or competitive athletics.^43,44^ Furthermore, the types of physical activity performed prior to BCT also affected MSKI risk in the sex-specific models, as those who had previously participated in multidirectional sports (eg, basketball, soccer, or field hockey) had reduced MSKI risk compared with those who participated in nonmultidirectional sports (eg, running, rowing, or biking). This finding is consistent with results of prior research^45,46,47^ indicating that multidirectional loading is associated with favorable cross-sectional bone geometry^48^ and muscle quality^47^ and lower tibial strains during walking with load carriage.^49^

#### Psychological Factors

A previous report within a subset of the current study population found that higher scores on grit and positive hardiness subscales at the start of BCT were associated with lower odds of self-reported injury risk, while trainees reporting high scores on negative hardiness had increased risk of injury during training.^50^ Those findings, in relationship to grit, contradict the results on medically reported MSKIs in this study and indicate that further investigation of the aspects (subscales) of grit is warranted. Hardiness (negative dimension) was associated with high MSKI risk in the female-specific model (Table 2) but not in the male-specific model, suggesting there may be sex-specific differences in the association between resilience metrics and MSKI risk.

#### Sleep Parameters

Consistent with prior research,^51,52^ these results highlight the importance of sleep in a training environment. Persons with poor sleep quality and insufficient sleep duration prior to BCT had an increased risk of MSKI.

### Clinical and Research Implications

When examining the total cohort MSKI risk model, it is important to consider all components collectively. For example, many studies have independently shown that higher BMI may increase the risk of MSKI.^4^ While the total cohort MSKI risk model showed a reduction in MSKI with higher BMI, this is in the context of body fat percentage (which is also in the model) remaining the same. Thus, an increased BMI would be due to an increase in fat-free mass.

Several interventions to reduce MSKI during BCT have been initiated.^53,54^ While some have focused on the period prior to BCT through a phased or delayed BCT entry program, most are initiated during BCT. The results of the present study suggest that a focused intervention to collectively increase physical activity, reduce body fat, reduce tobacco use, improve nutrition, improve psychological resilience, and promote positive sleep behaviors should begin in advance of novel training.

### Strengths and Limitations

The strengths of this study include its prospective design, comprehensive range of assessed risk factors, investigative statistics, and the model-building methods with internal cross-validation, application of the Youden index to create probability cut points of risk, and maximized measures of model performance and accuracy. While BCT provides a well-controlled field study environment, the voluntary nature of research can present inherent limitations, particularly when it comes to participant availability. Efforts were implemented to collect complete data from all participants, and appropriate statistics were used to account for missing data. Variable selection into the final models, particularly the sex-specific models, may have been affected by smaller sample sizes for some variable subgroups. In addition, self-report of some assessments was subject to participant disclosure. Additionally, there may be incomplete generalizability of results to nonmilitary populations.

Lastly, MSKI is a broad outcome, which may have prevented achieving higher NPV and PPV, sensitivity, specificity, and AUROC. Differing types of MSKI have different etiologies and potentially different injury factors. We also did not investigate changes in variables, such as body composition or bone density during BCT, and their relationship with MSKI. Follow-up analyses will test the hypotheses on changes in such variables, with the goal of building a comprehensive model inclusive of pre- and intra-BCT factors associated with MSKI risk.

## Conclusions

In this prospective cohort study of young Army trainees, a combination of 27 factors present at the start of BCT—factors in MSKI risk during training—were identified. Policies that apply a tiered quantification risk metric to guide screening practices and that incorporate multifactorial interventions during preparation for periods of intense physical activity may play a role in reduced MSKI risk.

## Notes

Supplement 1. **eMethods.** Overview of All Candidate Constructs and Their Variables, Measures by Conceptual Domain **eTable 1.** Model Candidate Constructs and Measures Considered for Final Models **eTable 2.** Total Cohort Model, Regression Model Details (SAS output) **eTable 3.** Descriptives for Those Variables in the Female- and Male-Specific MSKI Models **eResults.** eResults. Summary of the Total Cohort and Sex-Stratified (Female and Male) Models, Including Model Equations **eTable 4.** Female-Specific Model, Regression Model Details (SAS Output) **eTable 5.** Male-Specific Model, Regression Model Details (SAS Output)
eReferences

Supplement 2. **eAppendix.** Computation Spreadsheet Demonstrating Examples of Stoplight Model (Green, Amber, and Red) Risk Scenarios Presenting Computations for Green, Amber, and Red MSKI Risk Examples Described in the Article

Supplement 3. **Nonauthor Collaborators.** The ARIEM Reduction in Musculoskeletal Injury (ARMI) Study Team

Supplement 4.
Data Sharing Statement
