# Supplementary material for: Model for Musculoskeletal Injury Risk Factors Among US Army Basic Combat Trainees
Source: JAMA Netw Open. 2025 Jun 2;8(6):e2513177. doi: 10.1001/jamanetworkopen.2025.13177 (PMC12131099; doi:10.1001/jamanetworkopen.2025.13177)
Supplement: Supplement 1. — eMethods. Overview of All Candidate Constructs and Their Variables, Measures by Conceptual Domain eTable 1. Model Candidate Constructs and Measures Considered for Final Models eTable 2. Total Cohort Model, Regression Model Details (SAS output) eTable 3. Descriptives for Those Variables in the Female- and Male-Specific MSKI Models eResults. eResults. Summary of the Total Cohort and Sex-Stratified (Female and Male) Models, Including Model Equations eTable 4. Female-Specific Model, Regression Model Details (SAS Output) eTable 5. Male-Specific Model, Regression Model Details (SAS Output) eReferences [file jamanetwopen-e2513177-s001.pdf]

Supplemental Online Content

Foulis SA, Proctor SP, Spiering BA, et al; the ARIEM Reduction in Musculoskeletal Injury (ARMI) Study Team. Predictive model for musculoskeletal injury risk factors among US Army basic combat trainees. *JAMA Netw Open*. 2025;8(6):e2513177. doi:10.1001/jamanetworkopen.2025.13177

eMethods. Overview of All Candidate Constructs and Their Variables, Measures by Conceptual Domain

eTable 1. Model Candidate Constructs and Measures Considered for Final Models

eTable 2. Total Cohort Model, Regression Model Details (SAS output)

eTable 3. Descriptives for Those Variables in the Female- and Male-Specific MSKI Models

eResults. Summary of the Total Cohort and Sex-Stratified (Female and Male) Models, Including Model Equations

eTable 4. Female-Specific Model, Regression Model Details (SAS Output)

eTable 5. Male-Specific Model, Regression Model Details (SAS Output)

eReferences

This supplemental material has been provided by the authors to give readers additional information about their work.

© 2025 Foulis SA et al. *JAMA Network Open*

eMethods: Overview of all candidate constructs and their variables, measures by conceptual domain

We first chose seven conceptual domains that have been found to be relevant to MSKI outcomes in the BCT trainee environment. Within each domain, we considered several candidate variables based on prior research and conceptual models of the development and subsequent course of injury. Prior to analyses, we examined the correlations between all variables and excluded those variables from consideration, where correlations with related variables were .90 or higher. Excluded variables included: ‘DXA_Total_Mass’, ‘Sports PA’, and ‘Max_Vertec’ because each was highly correlated with ‘Weight’, ‘Load Class’, and Max_Vertec’, respectively.

Supplement eTable 1 below includes all the candidate constructs and their measures by conceptual domain for potential inclusion in the regression models as predictors.

Demographics considered included **age** (in years), **sex** (at birth), and **race and ethnicity.** For the latter, participants were asked to respond to the following question in the baseline questionnaire: “What is your racial background? (Select all that apply)”: White or Caucasian, Black or African American, Native American/Alaskan Indian, Indian or from Indian subcontinent, Asian, Native Hawaiian/Pacific Islander, Other. Also, they were asked to respond to “Are you Hispanic or Latino?”: yes or no. Their responses were collapsed by the investigators into the following categories for analyses: African American/Black Non-Hispanic; Asian Non-Hispanic; Hispanic; Other Non-Hispanic; White Non-Hispanic. These categories are what the Department of Defense uses on the Defense DD Form 96 to record demographics of incoming solider. (All who responded as ‘Hispanic’ were coded as such; those who responded to more than one category that did not include Hispanic were coded as ‘Other Non-Hispanic’. Those who responded as being from the Indian subcontinent were coded as ‘Asian’.) The “Other” race group is classified as those who define as multiracial, and those who define as Native American/Alaskan Native, Indian or from Indian Subcontinent, Native Hawaiian/Pacific Islander due to their sparse numbers in this cohort.

Body Composition Metrics considered included **height (cm)**, **weight (kg)**, **body mass index (BMI**,kg/m^2), and the following measures collected using dual-energy x-ray absorptiometry (DEXA, Hologic Horizon model A, Hologic, Inc., Marlborough, MA): **total mass (kg)**, **fat mass (kg)**, **percent body fat (%BF)**, **lean mass (kg)**, **total body mineral content (g, BMC), and total bone mineral density (g/cm2, BMD).**

Standing **heigh**t was measured to the nearest 0.1 cm, with participants standing in bare or stocking feet on a flat surface, feet together, knees straight, and the head, shoulder blades, buttocks, and heels in contact with the stadiometer. Body **weight** was measured using a calibrated electronic scale accurate to 0.1 kg. Participants were dressed in their physical training (PT) shorts and short-sleeved shirt without shoes or in similar attire. **BMI** was computed from height and weight measures[1].

© 2025 Foulis SA et al. *JAMA Network Open*

Total body estimates of **%BF, BMD, BMC, body content of total mass, and non-bone lean tissue** were determined via dual energy x-ray absorptiometry (DXA, Prodigy, GE Healthcare, Madison, WI) using manufacturer described procedures and supplied algorithms[2]. Precision of this measurement is better than ± 0.5% for %BF, and ±1.0% for total BMD. Whole body scanning from head to toe took approximately 3-5 minutes and a quality control phantom was scanned daily to ensure the machine was calibrated before each data collection session.

Nutritional Status Indicators considered were **Vitamin D** (25OHD (ng/mL), and iron and calcium status markers (namely, **iron** (ug/dL), **calcium** (mg/dL), **ionized calcium** (from the iSTAT (mmol/L)), **total iron binding capacity** (TIBC, ug/dL), and **ferritin** (ng/mL)).

These indicators were measured in fasting blood samples (approximately 35 mL per draw, approximately 70 mL total) collected from venous blood by a USARIEM credentialed phlebotomist. Per USARIEM policy, there were to be no more than 3 separate needle sticks per participant. Biological samples were analyzed on site for a small number of time and temperature sensitive markers. The rest of the samples were properly prepared, packaged and stored for shipping back to the USARIEM Central Lab for analysis or shipped to a commercial laboratory or academic institution on contract. In summary, depending on analyte, the markers were analyzed by ELISA methods, via Immulite, Multiplex, Dimension instrumentation, or similar standard laboratory assays.

Prior medical and health history measures considered included **current tobacco use, nonsteroidal anti-inflammatory drug (NSAID) use, history or a stress fracture, history of a broken bone, and history of concussion**.

These data were obtained from questions included in the baseline survey:

-Tobacco use: ("Prior to coming to BCT, were you ever a regular user of tobacco products?") yes/no
-NSAID use: A series of questions asked if 'In the 60 days prior to BCT, have you taken the following medication?" Acetaminophen, Aspirin, Ibuprofen, Naproxen, Indomethacin, Celecoxib, Meloxicam, Other/don't know; If the participant answered positive for any of the named NSAIDs, it was considered 'yes', otherwise 'no'.
-Stress Fracture provider: "Has a healthcare provider ever told you that you had a stress fracture?" yes/no
-Broken bone: "Have you ever broken a bone?" yes/no
-Concussion: "Have you ever been diagnosed with a concussion? A concussion is defined as any hit, bump or blow to the head that results in abnormal brain function." yes/no

Several additional questions were asked of female participants and considered for inclusion in the female-specific model: **Age at first period, History of amenorrhea, History of contraceptive use, Current contraceptive use**

© 2025 Foulis SA et al. *JAMA Network Open*

-Age at 1st period: “At what age did you have your first period?” Younger than 10 years old; 10-12 years old, 13-15 years old; Older than 15 years old
-History of amenorrhea: “Have your periods ever stopped for 3 consecutive months or longer (for reasons other than pregnancy or contraceptive use)?” No, never; Yes it has happened before; Yes, that is the situation currently. The latter two ‘yes’ responses were combined and coded as ‘yes’ for the analyses.
-History of contraception use: “Have you ever used hormonal contraceptives (e.g., the ‘pill’, patch, injection/shot, ring, implant, IUD) or copper IUD (e.g. Paragard))?” yes; no; I don’t know. If persons indicated a ‘don’t know’ answer, then they were coded as missing.
-Current contraception use: “Are you currently using hormonal contraceptives (e.g., the ‘pill’, patch, injection/shot, ring, implant, IUD) or copper IUD (e.g. Paragard))?” yes; no

Prior history of physical activity/fitness measures and health factors were obtained via survey questions that have been used in prior USARIEM research, primarily the Physical Demands Study and the Occupational Physical Assessment Test Study[3,4] (two studies designed to develop and validate the current Army’s physical assessment testing conducted as part of the Army recruiting process).

-Any participation in Sports prior to BCT? (‘Sports_PA’): ‘Have you ever played or participated in any sports/ regular physical activity?’ yes, no

-Sport Loading classification (‘LoadClass’): if participants answered yes to the ‘Sports_PA’ question above, they were asked to complete a checklist indicating what sports/physical activity they participated in. Each of the named sports or activities were *a priori* coded as a multidirectional, unidirectional, or non-impact sport. Multiaxial sports are defined as those in which the athlete is required to change directions frequently resulting to dynamic force loading to the legs (such as, soccer, basketball, rugby). Some examples of unidirectional sports are running, rowing, bicycling; non-impact sports examples include swimming and equestrian. For the analyses, the latter two were grouped and coded as non-multidirectional sports. If a participant reported no participation in sports prior to BCT on question ‘Sports_PA’, then ‘LoadClass’ was coded as 1; If a participant participated in sports but not a multidirectional one, then ‘LoadClass’ was coded as 2; if a participant indicated playing any multidirectional sport, LoadClass’ was coded as multidirectional=3 and was the reference.

-**Exercise per week** in 2 months prior to BCT (‘Exercise’): “Over the past 2 months, prior to entering BCT, what was the average number of times per week you exercised or played sports for at least 30 min at a time?” Never; 1 time per week; 2 times per week; 3 times per week; 4 times per week; 5 times per week; 6 times per week; 7 or more times per week. For analyses, recoded 3 categories: *0-2x per week, 3-4x per week, 5 or more times per week (reference).

-How does **Exercise** compare to exercise over prior year (‘YrExercise’): ‘Compare to your exercise or sports frequency in past 2 months to that during the entire year prior to

entering BCT?” much less; somewhat less; about the same; somewhat more; much more. For analyses: coded each response 1-5, with 3 the reference.

-**Physical Activity** level prior to BCT (‘PA_Level’): “Compared to others your same age and sex, how would you rate yourself as to the amount of physical activity you performed prior to entering BCT?” much less active; somewhat less active; about the same; somewhat more active; much more active. For analyses: coded each response 1-5, with 3 the reference.

-**Running**/jog per week in 2 months prior to BCT (‘RunWk’): “Over the past 2 months, prior to entering BCT, how many times per week did you run or jog?” Never; 1 time per week; 2 times per week; 3 times per week; 4 times per week; 5 times per week; 6 times per week; 7 or more times per week. For analyses recoded 3 categories: *0-2x per week, 3-4x per week, 5 or more times per week (reference).

-**Weight Training** per week in 2 months prior to BCT (‘WeightWk’): “Over the past 2 months, prior to entering BCT, how many times per week did you perform weight training exercises (with barbells, kettlebells, dumbells, machines, etc.)?” Never; 1 time per week; 2 times per week; 3 times per week; 4 times per week; 5 times per week; 6 times per week; 7 or more times per week. For analyses recoded 3 categories: *0-2x per week, 3-4x per week, 5 or more times per week (reference).

Several objective measures were included. These included **maximum vertical jump (cm)** and **average of vertical jump height (cm)**,measures of Lower Extremity Strength/Power),assessed using the Vertec, (Sports Imports, Hilliard, OH, USA)[5]. For this test, while wearing physical training uniforms, recruits jump as high as possible while standing next to a calibrated device designed to measure jump height (Vertec jump training system,). Recruits are given 2 practice attempts, followed by 3 trials for record. Average height of the best 2 of 3 jumps (‘Vertec_CM’) and maximum vertex score (cm), ‘Max Vertec Score’) were considered for analysis.

Also, Occupational Performance Assessment Test (OPAT) scores determined at entry into the service were obtained from the Army trainees’ records, with their consent. The OPAT Test scores included were the **Power Throw (PT) Distance** (cm), **Deadlift** (kg), **Long Jump distance** (cm), and **Interval Aerobic Run** (IR) or beep test (number of levels completed (‘Shut’), total number of shuttles recorded (IR_Tot_Shut)[3].

Mental performance metrics of resilience, assessed using the Angela Duckworth Grit Scale[6] and the Dispositional Resilience Scale –II[7], provide a **total grit score** (along with subscale scores reflecting ambition, consistency, and perseverance) and **positive hardiness and negative hardiness scores**, respectively. Also, we include the Pain Catastrophizing Scale[8] which yielded the **total pain catastrophizing**. The (PCS) is a 13-item measure of an individual’s negative thoughts and feelings during pain, assessing the three dimensions of catastrophizing (rumination, magnification, and helplessness) using a 5-point Likert scale (0 = not at all, 4 = all the time), with total scores ranging from 0 to 52. Higher scores reflect higher levels of catastrophizing. The PCS is psychometrically sound and possesses high internal consistency, concurrent, criterion-related, and discriminant validity[9].

© 2025 Foulis SA et al. *JAMA Network Open*

Sleep parameters included metrics from the Pittsburgh Sleep Quality Index[10] (**Global sleep score**) and ratings of per BCT **Sleep Quality**, **Sleep Duration**, and **Sleep Efficiency**. The PSQI asks about sleep characteristics over the past 1 month. It consists of 18 items that are grouped and scored into seven component categories: subjective sleep quality, sleep latency, sleep duration, habitual sleep efficiency, sleep disturbances, use of sleep medications, and daytime dysfunction. The Global sleep score is a continuous measure which is a sum of all the component scores.

-Sleep Duration ('Duration'): "In the month prior to BCT, please give an estimate of the number of hours of sleep on average you got a night? (This may be different from the number of hours you spent in bed.)" Each was response was then categorized and coded as >7 hours=0; 6-7 hours=1; 5-6 hours=2; <5 hours=3, with 0 coded as the reference.

-Sleep Quality ('SleepQual'): "In the month prior to BCT, how would you rate your sleep quality overall?" very good; fairly good; fairly bad; very bad. Coded as 0 to 3 with 0 the reference.

-Sleep efficiency ('Eff_cat') is computed into a percentage score using responses to the number of hours slept and number of hours spent in bed (determined from questions about what time participant went to bed and go up in the morning). Each response was then then categorized and coded as >85%=0; 75-84%=1; 65-74%=2; <65%=3, with 0 coded as the reference.

© 2025 Foulis SA et al. *JAMA Network Open*

eTable 1: Model candidate constructs and measures considered for final models

Category	Variable	Variable Abbreviation	Value Labels and Coding	Response Type	Source
**Demographics**	Age (years) (missing 7)	'Age'		Continuous	Survey
	Sex	'Sex'	(0)Male<br>(1)Female	Binary	Survey
	Race/ethnicity (missing 25)	'Race'	(1) African American/Black, Non-Hispanic<br>(2) Asian, Non-Hispanic<br>(3) Hispanic<br>(4) Other*, Non-Hispanic<br>(5) White, Non-Hispanic	Categorical	Survey
**Anthropometrics and Body Composition**	Weight (kg) (missing 31)	'Weight'		Continuous	Measured
	Height (cm) (missing 16)	'Height'		Continuous	Measured
	BMI (kg/m2) (missing 31)	'BMI'	(1) <18.5<br>(2) 18.5 - 24.9<br>(3) 25-29.9<br>(4) 30+	Categorical	Measured (computed from Height and Weight)
	DXA Bodyfat (%) (missing 174)	'BodyFat'		Continuous	Measured
	DXA Lean Mass (kg) (missing 174)	'Leanmass'		Continuous	Measured
	DXA TBLH_BMC (g) (missing 174)	'BMC'		Continuous	Measured
	DXA TBLH_BMD (g/cm2) (missing 175)	'BMD'		Continuous	Measured
**Nutritional Status Indicators**	Vitamin D (25OH, ng/mL) (missing 275)	'VitD'		Continuous	Measured
	Iron (ug/dL) (missing 258)	'Iron'		Continuous	Measured
	Calcium (mg/dL) (missing 453)	'Ca'		Continuous	Measured
	Ionized Calcium ISTAT (mmol/L) (missing 280)	'iStat_iCa'		Continuous	Measured
	Total Iron Binding Capacity (ug/dL) (missing 275)	'TIBC'		Continuous	Measured
	Ferritin (ng/mL) (missing 420)	'Ferritin'		Continuous	Measured

© 2025 Foulis SA et al. *JAMA Network Open*

eTable 1: Model candidate constructs and measures (continued)

Category	Variable	Variable Abbreviation	Value Labels and Coding	Category	Variable
**Medical and Health History**	Tobacco Use (missing 25)	'Tobacco'	(1)Yes<br>(0)No	Binary	Survey
	NSAID Usage (missing 83)	'NSAID Ever'	(1)Yes<br>(0)No	Binary	Survey
	History of Stress Fracture (missing 11)	'SF'	(1)Yes<br>(0)No	Binary	Survey
	History of Broken Bone (missing 11)	'BB'	(1)Yes<br>(0)No	Binary	Survey
	History of Concussion (missing 11)	'Concussion'	(1)Yes<br>(0)No	Binary	Survey
[Females Only]	Age of First Period (missing 34)	'Period_Age '	(1) <10 Years Old<br>(2) 10-12 Years Old<br>(3) 13-15 Years old<br>(4) >15 Years Old	Categorical	Survey
	History of Amenorrhea (missing 42)	'Ever_Ameno'	(1)Yes<br>(0)No	Binary	Survey
	History of Contraceptive Use (missing 34)	'Ever_Contra_Use'	(1)Yes<br>(0)No	Binary	Survey
	Current Contraceptive Use (missing 32)	'Current_Contra_Use'	(1)Yes<br>(0)No	Binary	Survey

© 2025 Foulis SA et al. *JAMA Network Open*

eTable 1: Model candidate constructs and measures (continued)

Category	Variable	Variable Abbreviation	Value Labels and Coding	Category	Variable
**History of Sports and Past/Current Physical Activity/Fitness**	Sport Loading Classification (missing 11)	'LoadClass'	(1)Never Played Sports<br>(2)Played Non-Multidirectional Sports<br>(3)Played Multidirectional Sports	Categorical	Survey
	Exercise frequency per week in 2 months before BCT (missing 43)	'Exercise'	(1) Never or 1-2 times/wk<br>(2) 3-4 times/wk<br>(3) 5-7+ times/wk	Categorical	Survey
	Exercise in the Year Prior to BCT, compared to 2 months before BCT (missing 53)	'ExYear'	(1) Much less<br>(2) Somewhat less<br>(3) About the same<br>(4) Somewhat more<br>(5) Much more	Categorical	Survey
	Physical Activity Level Pre-BCT (missing 43)	'PA_Level'	(1) Much less active<br>(2) Somewhat less active<br>(3) About the same<br>(4) Somewhat more active<br>(5) much more active	Categorical	Survey
	Running (missing 58)<br>"Over the last 2mo prior to BCT, how many times per week did you run or jog?"	'Running'	(1) 0-2 times/wk<br>(2) 3-4 times/wk<br>(3) 5+ times/wk	Categorical	Survey

© 2025 Foulis SA et al. *JAMA Network Open*

eTable 1: Model candidate constructs and measures (continued)

Category	Variable	Variable Abbreviation	Value Labels and Coding	Category	Variable
**History of Sports and Past/Current Physical Activity/Fitness (Continued)**	Weight Training (missing 55) "Over the last 2mo prior to BCT, how often did you perform weight training exercises?"	'WeightsWk'	(1) 0-2 times/wk<br>(2) 3-4 times/wk<br>(3) 5+ times/wk	Categorical	Survey
	Vertec Average (cm) (missing 343)	'Vertex_CM'		Continuous	Measured
	OPAT Deadlift (kg) (missing 153)	'DL'		Continuous	Army Records
	OPAT Long Jump Distance (cm) (missing 153)	'LJDist'		Continuous	Army Records
	OPAT Power Throw Distance (cm) (missing 153)	'PTDist'		Continuous	Army Records
	OPAT Interval Aerobic Run: Number of Levels Completed (missing 153)	'Shut'		Continuous	Army Records
	OPAT Interval Aerobic Run: Total Number of Shuttles Completed (missing 153)	'IR_Tot_Shut'		Continuous	Army Records
**Psychological Factors**	Grit: Summary score (missing 302)	'Grit_sum'		Continuous	Survey
	Hardiness: Positive summary score (missing 294)	'Hardiness PosSum'		Continuous	Survey
	Hardiness: Negative summary score (missing 265)	'Hardiness NegSum'		Continuous	Survey
	Total Pain Catastrophizing score (missing 448)	'Total_PCS'		Continuous	Survey

© 2025 Foulis SA et al. *JAMA Network Open*

eTable 1: Model candidate constructs and measures (continued)

Category	Variable	Variable Abbreviation	Value Labels and Coding	Category	Variable
**Sleep Parameters**	PSQI Global Sleep Score (missing 109)	'PSQIGlobal'		Continuous	Survey
	PSQI Pre-BCT Sleep Duration (missing 109)	'Duration'	(0) >7 Hours<br>(1) 6-7 Hours<br>(2) 5-6 Hours<br>(3) <5 Hours	Categorical	Survey
	PSQI Pre-BCT Sleep Quality (missing 127)	'SleepQual'	(0) Very Good<br>(1) Fairly Good<br>(2) Fairly Bad<br>(3) Very Bad	Categorical	Survey
	PSQI Pre-BCT Sleep Efficiency (missing 109)	'Eff_cat'	(0) >84.5%<br>(1) 74-5-84.49%<br>(2) 65-74.49%<br>(3) <65%	Categorical	Survey

***Other=** those non-Hispanic participants who define themselves as multiracial, and those who define themselves as Native American/Alaskan Native, Indian or from Indian Subcontinent, or Native Hawaiian/Pacific Islander

© 2025 Foulis SA et al. *JAMA Network Open*

eTable 2: Total cohort model, regression model details (SAS output)

Parameter	Class	Estimate	Std Error	OddsRatioEst	LowerCL	UpperCL
Intercept		-0.0094	0.0366	0.99	.	.
Age		0.0552	0.0117	1.06	1.03	1.08
Sex	0	.	.	.	.	.
Sex	1	0.2528	0.1606	1.29	0.94	1.77
Race	1	0.4079	0.1338	1.50	1.16	1.95
Race	2	0.1201	0.2058	1.13	0.75	1.69
Race	3	0.1838	0.1114	1.20	0.97	1.50
Race	4	0.3016	0.1498	1.35	1.01	1.81
Race	5	.	.	.	.	.
BMI	1	0.4650	0.3290	1.59	0.84	3.03
BMI	2	.	.	.	.	.
BMI	3	-0.1975	0.1082	0.82	0.66	1.01
BMI	4	-0.1782	0.1992	0.84	0.57	1.24
BodyFat		0.0450	0.0095	1.05	1.03	1.07
BMC		-0.00005	0.0002	1.00	1.00	1.00
BMD		-1.6067	0.7007	0.20	0.05	0.80
VitD		-0.0017	0.0049	1.00	0.99	1.01
Iron		-0.0012	0.0014	1.00	1.00	1.00
Ferritin		-0.0015	0.0007	1.00	1.00	1.00
Tobacco	0	.	.	.	.	.
Tobacco	1	0.1979	0.0973	1.22	1.01	1.47
SF	0	.	.	.	.	.
SF	1	0.2607	0.2287	1.30	0.83	2.03
BB	0	.	.	.	.	.
BB	1	0.3081	0.1049	1.36	1.11	1.67
Concussion	0	.	.	.	.	.
Concussion	1	0.0741	0.1310	1.08	0.83	1.39
Exercise	1	0.2112	0.1512	1.24	0.92	1.66
Exercise	2	0.1757	0.1100	1.19	0.96	1.48
Exercise	3	.	.	.	.	.
ExYear	1	-0.0040	0.1243	1.00	0.78	1.27
ExYear	2	0.0169	0.1174	1.02	0.81	1.28
ExYear	3	.	.	.	.	.
ExYear	4	0.1350	0.1203	1.14	0.90	1.45
ExYear	5	0.0296	0.1352	1.03	0.79	1.34
PALevel	1	0.1349	0.1585	1.14	0.84	1.56

© 2025 Foulis SA et al. *JAMA Network Open*

eTable 2: Total cohort model, regression model details (SAS output) (Continued)

Parm	Class	Estimate	Std Error	OddsRatioEst	LowerCL	UpperCL
PALevel	2	0.2442	0.1177	1.28	1.01	1.61
PALevel	3	.	.	.	.	.
PALevel	4	-0.0243	0.1160	0.98	0.78	1.23
PALevel	5	0.2065	0.1563	1.23	0.90	1.67
RunWk	1	0.1932	0.1049	1.21	0.99	1.49
RunWk	2	.	.	.	.	.
RunWk	3	0.0501	0.1096	1.05	0.85	1.30
WeightsWk	1	0.2146	0.1016	1.24	1.02	1.51
WeightsWk	2	.	.	.	.	.
WeightsWk	3	0.1610	0.1170	1.17	0.93	1.48
LJDistance		-0.0029	0.0016	1.00	0.99	1.00
PTDist		0.0014	0.0005	1.00	1.00	1.00
GritSum		0.0172	0.0071	1.02	1.00	1.03
HardinessNegSum		0.0061	0.0069	1.01	0.99	1.02
TotalPCS		0.0028	0.0047	1.00	0.99	1.01
PSQIGlobal		0.0764	0.0226	1.08	1.03	1.13
Duration	0	.	.	.	.	.
Duration	1	-0.2213	0.0971	0.80	0.66	0.97
Duration	2	-0.1983	0.1752	0.82	0.58	1.16
Duration	3	0.1111	0.2178	1.12	0.73	1.71
SleepQual	0	.	.	.	.	.
SleepQual	1	-0.0961	0.1068	0.91	0.74	1.12
SleepQual	2	-0.3616	0.1624	0.70	0.51	0.96
SleepQual	3	0.0329	0.2786	1.03	0.60	1.78

Std Error= Standard Error
LowerCL= Lower limit of 95% Confidence Interval of Odds Ratio
UpperCL= Upper limit of 95% Confidence Interval of Odds Ratio

© 2025 Foulis SA et al. *JAMA Network Open*

eTable 3: Descriptives for those variables in the male and female MSKI models

	Variable	Description/Coding	Total Population (N=2,988)	Males (N=1,880)	Females (N=1,108)
**Demographics**	Age (yrs) (median (IQR))	'Age'	19.00 (4.00)	20.00 (4.00)	19.00 (4.00)
	Sex (N(%))	Male ('Sex_0' (ref))<br>Female ('Sex_1')	1,880 (62.92%)<br>1,108 (37.08%)		
	Race/ethnicity (N(%))	Asian, Non-Hispanic ('$Race_1$')<br>Black, Non-Hispanic ('$Race_2$')<br>Hispanic ('$Race_3$')<br>Other*, Non-Hispanic ('$Race_4$')<br>White, Non-Hispanic ('$Race_5$' (ref))	134 (4.52%)<br>620 (20.92%)<br>705 (23.79%)<br>264 (8.91%)<br>1,240 (41.85%)	75 (4.02%)<br>322 (17.26%)<br>418 (22.40%)<br>161 (8.63%)<br>890 (47.70%)	59 (5.38%)<br>298 (27.16%)<br>287 (26.16%)<br>103 (9.39%)<br>350 (31.91%)
**Anthropometric and Body Composition**	BMI (kg/m2) (N(%))	<18.5 ('BMI_1')<br>18.5 - 24.9 ('BMI_2(ref)')<br>25-29.9 ('BMI_3')<br>30+ ('BMI_4')	47 (1.59%)<br>1,515 (51.23%)<br>1,176 (39.77%)<br>219 (7.41%)	31 (1.67%)<br>868 (46.79%)<br>756 (40.75%)<br>200 (10.78%)	16 (1.45%)<br>647 (58.71%)<br>420 (38.11%)<br>19 (1.72%)
	DXA Bodyfat (%) (M (SD))	'BodyFat'	26.57 (±7.63)	23.11 (±6.67)	32.28 (±5.35)
	DXA Bone Mineral Content (BMC) (g) (M (SD))	'BMC'	2,763.50 (±511.76)	3,016.60 (±438.24)	2,345.33 (±310.82)
	DXA Bone Mineral Density (BMD) (g/cm2) (M (SD))	'BMD'	1.24 (±0.14)	1.28 (±0.14)	1.18 (±0.11)
**Nutritional Status Indicators**	Vitamin D (25OH, ng/mL) (M (SD))	'VitD'	24.34 (±8.96)	24.48 (±8.86)	24.09 (±9.13)
	Iron (ug/dL) (M (SD))	'Iron'	71.84 (±32.12)	79.13(±30.41)	59.55 (±31.19)
	Total Iron Binding Capacity (ug/dL) (M (SD))	'TIBC'	306.13 (±50.12)	291.97 (±40.77)	329.86 (±55.13)
	Ferritin (ng/mL) (median (IQR))	'Ferritin'	79.40 (95.45)	113.50 (87.90)	30.80 (31.45)
**Medical and Health History**	Tobacco Use (N(%))	Yes ('$Tobacco_1$')<br>No ('$Tobacco_0$' (ref))	698 (23.56%)<br>2,265 (76.44%)	513 (27.45%)<br>1,356 (72.55%)	185 (16.91%)<br>909 (83.09%)
	History of Stress Fracture (N(%))	Yes ('SF_1')<br>No ('SF_0' (ref))	97 (3.32%)<br>2,828 (96.68%)	55 (2.99%)<br>1,783 (97.01%)	42 (3.86%)<br>1,045 (96.14%)
	History of Broken Bone (N(%))	Yes ('BB_1')<br>No ('BB_0' (ref))	592 (20.18%)<br>2,341 (79.82%)	427 (23.18%)<br>1,415 (76.82%)	165 (15.12%)<br>926 (84.88%)

© 2025 Foulis SA et al. *JAMA Network Open*

eTable 3: Descriptives for those variables in the male and female MSKI models (Continued)

	Variable	Description/Coding	Total Population (N=2,988)	Males (N=1,880)	Females (N=1,108)
History of Sports and Past/Current Physical Activity/Fitness	Sports Loading Classification (N (%))	Never Played sports (‘LoadClass$_1$’)	463 (15.55%)	291 (15.52%)	172 (15.61%)
		Played non-multiaxial sports (‘LoadClass$_2$’)	213 (7.15%)	118 (6.29%)	95 (8.62%)
		Played multiaxial sports (‘LoadClass$_3$’(ref))	2,301 (77.29%)	1,466 (78.19%)	835 (75.77%)
	Exercise frequency per week in 2 months before BCT (N (%))	0-2 times/week (‘Exercise$_1$’)	1068 (35.74%)	604 (32.56%)	464 (42.57)
		3-4 times/week (‘Exercise$_2$’)	1066 (35.68%)	680 (36.66%)	386 (35.41)
		5+ times/week (Exercise3’(ref))	811 (27.14%)	571 (30.78%)	240 (22.02)
	Exercise in the Year Prior to BCT, compared to 2 months before BCT (N(%))	Much less (‘ExYear$_1$’)	536 (18.26%)	313 (16.89%)	223 (20.61%)
		Somewhat less (‘ExYear$_2$’)	575 (19.59%)	340 (18.35%)	235 (21.72%)
		About the same (‘ExYear$_3$’(ref))	914 (31.14%)	636 (34.32%)	278 (25.69%)
		Somewhat more (‘ExYear$_4$’)	521 (17.75%)	329 (17.75%)	192 (17.74%)
		Much more (‘ExYears$_5$’)	389 (13.25%)	235 (12.66%)	154 (14.23%)
	Physical Activity Level, “Compared to others your same age and sex, how would you rate yourself on the level of physical activity prior to BCT? (N (%))	Much less Active (‘PA_Level$_1$’)	345 (11.71%)	188 (10.13%)	157 (14.42%)
		Somewhat less active(‘PA_Level$_2$’)	756 (25.67%)	413 (22.25%)	343 (31.50%)
		About the same (‘PA_Level3’(ref))	683 (23.19%)	437 (23.55%)	246 (22.59%)
		Somewhat more active (‘PA_Level$_4$’)	805 (27.33%)	556 (29.36%)	249 (22.87%)
		Much more active (‘PA_Level$_5$’)	356 (12.09%)	262 (14.12%)	94 (8.63%)
	Running, "Over the last 2mo prior to BCT, how many times per week did you run or jog?" (N (%))	0-2 times/week (‘RunWk$_1$’)	1,640 (54.89%) 977	996 (53.90%)	644 (59.19%)
		3-4 times/week (‘RunWk2’ (ref))	(32.70%)	637 (34.47%)	340 (31.42%)
		5+times/week (‘RunWk$_3$’)	313 (10.47%)	215 (11.63%)	98 (9.06%)
	Weight Training, "Over the last 2mo prior to BCT, how often did you perform weight training exercises?" (N (%))	0-2 times/week (‘WeightsWk$_1$’)	1826 (61.11%)	1,069 (57.88%)	757 (69.71%)
		3-4 times/week (‘WeightsWk2’ (ref))	713 (23.86%)	474 (24.66%)	239 (22.01%)
		5+ times/week (WeightsWk$_3$’)	394 (13.19%)	304 (16.46%)	90 (8.29%)
	OPAT Power Throw Distance (cm) (M (SD))	‘PTDIst’	504.06 (±102.20)	509.60 (±102.74)	494.72 (±100.64

© 2025 Foulis SA et al. *JAMA Network Open*

eTable 3: Descriptives for those variables in the male and female MSKI models (Continued)

	Variable	Description/Coding	Total Population (N=2,988)	Males (N=1,80)	Females (N=1,108)
**Psychological Factors**	Total Grit Summary Score (M (SD))	‘Grit_sum’	42.34 (±6.52)	42.06 (±6.44)	42.81 (±6.63)
	Hardiness: Negative summary score (M (SD)	‘Hardiness NegSum’	29.22 (±6.85)	29.41 (±6.91%)	28.92 (±6.75)
	Total Pain Catastrophizing score (median (IQR))	‘Total_PCS’	8.00 (14.00)	8.00 (13.00)	9.00 (16.00)
**Sleep Parameters**	PSQI Global Sleep Score (median (IQR))	‘PSQIGlobal’	4.00 (3.00)	4.00 (3.00)	3.00 (4.00)
	PSQI-Sleep Duration, Pre-BCT (N (%))	>7 Hours (‘Duration0’(ref))<br>6-7 Hours (‘Duration$_1$’)<br>5-6 Hours (‘Duration$_2$’)<br><5 Hours (‘Duration$_3$’)	1,626 (56.48%)<br>901 (31.30%)<br>212 (7.36%)<br>140 (4.86%)	1,012 (55.94%)<br>580 (32.06%)<br>123 (6.80%)<br>94 (5.20%)	614 (57.38%)<br>321 (30.00%)<br>89 (8.32%)<br>46 (4.30%)
	PSQI-Sleep Quality, Pre-BCT (N (%))	Very good (‘Sleepqual0’(ref))<br>Fairly good (‘SleepQual$_1$’)<br>Fairly bad (‘SleepQual$_2$’)<br>Very bad (‘SleepQual$_3$’)	716 (25.03%)<br>1,621 (56.66%)<br>432 (15.10%)<br>92 (3.22%)	447 (24.87%)<br>1,030 (57.32%)<br>266 (14.80%)<br>54 (3.01%)	269 (25.28%)<br>591 (55.55%)<br>166 (15.60%)<br>38 (3.57%)

*Other= those non-Hispanic participants who define themselves as multiracial, and those who define themselves as Native American/Alaskan Native, Indian or from Indian Subcontinent, or Native Hawaiian/Pacific Islander

© 2025 Foulis SA et al. *JAMA Network Open*

eResults:

Total cohort risk model Equation

$$
\begin{aligned}
&\mathrm{Logit}(\Pr[\mathrm{MSKI}]_{TotalCohort}) = -0.0094+0.0552*\mathrm{Age}+0.2528*\mathrm{Sex}_1+0.4079*\mathrm{Race}_1+\\
&0.1201*\mathrm{Race}_2+0.1838*\mathrm{Race}_3+0.3016*\mathrm{Race}_4+0.465*\mathrm{BMI}_1-0.1975*\mathrm{BMI}_3-0.1782*\mathrm{BMI}_4+\\
&0.045*\mathrm{BodyFat}-0.0000505*\mathrm{BMC}-1.6067*\mathrm{BMD}-0.0017*\mathrm{VitD}-0.0012*\mathrm{Iron}-\\
&0.0015*\mathrm{Ferritin}+0.1979*\mathrm{Tobacco}_1+0.2607*\mathrm{SF}_1+0.3081*\mathrm{BB}_1+0.0741*\mathrm{Concussion}_1+\\
&0.2112*\mathrm{Exercise}_1+0.1757*\mathrm{Exercise}_2-0.004*\mathrm{ExYear}_1+0.0169*\mathrm{ExYear}_2+0.135*\mathrm{ExYear}_4+\\
&0.0296*\mathrm{ExYear}_5+0.1349*\mathrm{PALevel}_1+0.2442*\mathrm{PALevel}_2-0.0243*\mathrm{PALevel}_4+0.2065*\mathrm{PALevel}_5\\
&+0.1932*\mathrm{RunWk}_1+0.0501*\mathrm{RunWk}_3+0.2146*\mathrm{WeightsWk}_1+0.161*\mathrm{WeightsWk}_3-\\
&0.0029*\mathrm{LJDistance}+0.0014*\mathrm{PTDist}+0.0172*\mathrm{GritSum}+0.0061*\mathrm{HardinessNegSum}+\\
&0.0028*\mathrm{TotalPCS}+0.0764*\mathrm{PSQIGlobal}-0.2213*\mathrm{Duration}_1-0.1983*\mathrm{Duration}_2+\\
&0.1111*\mathrm{Duration}_3-0.0961*\mathrm{SleepQual}_1-0.3616*\mathrm{SleepQual}_2+0.0329*\mathrm{SleepQual}_3
\end{aligned}
$$

Sex-stratified models (female and male) Equation and summaries

$$
\begin{aligned}
&\mathrm{Logit}(\Pr[\mathrm{MSKI}]_{female}) = +0.6541+0.0411*\mathrm{Age}+0.4052*\mathrm{Race}_1+0.5148*\mathrm{Race}_2+\\
&0.236*\mathrm{Race}_3+0.4004*\mathrm{Race}_4+0.5997*\mathrm{BMI}_1-0.3451*\mathrm{BMI}_3+0.9306*\mathrm{BMI}_4+0.0476*\mathrm{BodyFat}-\\
&0.001*\mathrm{BMC}+0.0031*\mathrm{TIBC}+0.2924*\mathrm{BB}_1-0.4015*\mathrm{LoadClass}_1+0.4231*\mathrm{LoadClass}_2+\\
&0.4721*\mathrm{Exercise}_1+0.4395*\mathrm{Exercise}_2-0.0998*\mathrm{ExYear}_1-0.0842*\mathrm{ExYear}_2+0.1456*\mathrm{ExYear}_4-\\
&0.4029*\mathrm{ExYear}_5+0.1016*\mathrm{PALevel}_1+0.22*\mathrm{PALevel}_2-0.0419*\mathrm{PALevel}_4+0.2874*\mathrm{PALevel}_5+\\
&0.3596*\mathrm{RunWk}_1+0.304*\mathrm{RunWk}_3+0.0017*\mathrm{PTDist}+0.0174*\mathrm{HardinessNegSum}+\\
&0.0849*\mathrm{PSQIGlobal}-0.0898*\mathrm{SleepQual}_1-0.5994*\mathrm{SleepQual}_2+0.1217*\mathrm{SleepQual}_3
\end{aligned}
$$

$$
\begin{aligned}
&\mathrm{Logit}(\Pr[\mathrm{MSKI}]_{male}) = -0.3922+0.0569*\mathrm{Age}+0.4587*\mathrm{Race}_1-0.1258*\mathrm{Race}_2+0.165*\mathrm{Race}_3+\\
&0.3205*\mathrm{Race}_4+0.0381*\mathrm{BodyFat}-1.8473*\mathrm{BMD}-0.0027*\mathrm{VitD}-0.0032*\mathrm{TIBC}-\\
&0.0017*\mathrm{Ferritin}+0.247*\mathrm{Tobacco}_1+0.4304*\mathrm{SF}_1+0.3407*\mathrm{BB}_1+0.0388*\mathrm{LoadClass}_1+0.177*\mathrm{LoadClass}_2+\\
&0.0769*\mathrm{ExYear}_1+0.0371*\mathrm{ExYear}_2+0.1394*\mathrm{ExYear}_4+0.2629*\mathrm{ExYear}_5+0.211*\mathrm{PALevel}_1+\\
&0.2104*\mathrm{PALevel}_2+0.0017*\mathrm{PALevel}_4+0.1211*\mathrm{PALevel}_5+0.165*\mathrm{RunWk}_1-\\
&0.0342*\mathrm{RunWk}_3+0.3184*\mathrm{WeightsWk}_1+0.0869*\mathrm{WeightsWk}_3+0.0235*\mathrm{GritSum}+0.0088*\\
&\mathrm{TotalPCS}+0.0362*\mathrm{PSQIGlobal}-0.2526*\mathrm{Duration}_1-0.243*\mathrm{Duration}_2+0.3984*\mathrm{Duration}_3
\end{aligned}
$$

In the female model, compared to the total cohort model, the additional variables of higher TIBC and sports load class (playing a unidirectional or non-impact sport compared to multidirectional sport) predicted increased MSKI risk. BMD, Iron, ferritin, vitamin D, prior stress fracture, history of concussion, tobacco use, frequency of weight training prior to BCT, vertical jump height, OPAT long jump distance, grit, pain catastrophizing, and ratings of sleep duration did not meet model inclusion criteria.

© 2025 Foulis SA et al. *JAMA Network Open*

In the male-specific model, compared to the total cohort model, additional risk factors included lower TIBC and load class (both playing a unidirectional or non-impact sport or no sports compared to multidirectional sport). However, BMI, BMC, iron, exercise frequency in 2 months prior to BCT, vertical jump height, OPAT long jump, OPAT power throw, negative hardiness, and ratings of sleep quality did not meet model inclusion criteria.

Comparing the sex-specific models, in terms of which variables are included, we find that the female-specific model included 'BMI', 'Exercise', 'PTDist', 'HardinessNegSum', and 'SleepQual', while the male-specific model did not. The male-specific model included 'BMD', VitD', 'Ferritin', 'Tobacco', 'SF', 'WeightsWk', 'Grit, 'TotalPCS', and 'SleepDuration', while the female-specific model did not.

Both the female- and male-specific models had somewhat lower ROC values (0.68 and 0.66 respectively) compared to the total cohort model (0.70).

The Youden's index cutpoint (medium risk threshold) resulted in slightly reduced sensitivity and specificity in the female model, compared to the total cohort model (see details in Table 3). For the female- and male-specific models, compared to the total cohort model, the sensitivity was reduced but each had somewhat higher specificity. The male-specific model cutpoints provided lower PPV and higher NPV, and the female-specific model cutpoints produced a higher PPV and lower NPV, compared to the total cohort model.

eTable 4: Female-specific model, regression model details (SAS output)

Parm	Class	Estimate	Std Error	OddsRatioEst	LowerCL	UpperCL
Intercept		0.6541	0.0633	1.92	.	.
Age		0.0411	0.0199	1.04	1.00	1.08
Race	1	0.4052	0.1876	1.50	1.04	2.17
Race	2	0.5148	0.3499	1.67	0.84	3.32
Race	3	0.2360	0.1854	1.27	0.88	1.82
Race	4	0.4004	0.2575	1.49	0.90	2.47
Race	5	.	.	.	.	.
BMI	1	0.5997	0.6802	1.82	0.48	6.91
BMI	2	.	.	.	.	.
BMI	3	-0.3451	0.1769	0.71	0.50	1.00
BMI	4	0.9306	0.6823	2.54	0.67	9.66
BodyFat		0.0476	0.0173	1.05	1.01	1.09
BMC		-0.0010	0.0003	1.00	1.00	1.00
TIBC		0.0031	0.0013	1.00	1.00	1.01
BB	0	.	.	.	.	.
BB	1	0.2924	0.1971	1.34	0.91	1.97
LoadClass	1	-0.4015	0.1993	0.67	0.45	0.99
LoadClass	2	0.4231	0.2589	1.53	0.92	2.54
LoadClass	3	.	.	.	.	.
Exercise	1	0.4721	0.2527	1.60	0.98	2.63
Exercise	2	0.4395	0.1813	1.55	1.09	2.21
Exercise	3	.	.	.	.	.
ExYear	1	-0.0998	0.2249	0.91	0.58	1.41
ExYear	2	-0.0842	0.2083	0.92	0.61	1.38
ExYear	3	.	.	.	.	.
ExYear	4	0.1456	0.2154	1.16	0.76	1.76
ExYear	5	-0.4029	0.2347	0.67	0.42	1.06
PALevel	1	0.1016	0.2601	1.11	0.66	1.84
PALevel	2	0.2200	0.1952	1.25	0.85	1.83
PALevel	3	.	.	.	.	.
PALevel	4	-0.0419	0.2031	0.96	0.64	1.43
PALevel	5	0.2874	0.2867	1.33	0.76	2.34
RunWk	1	0.3596	0.1811	1.43	1.00	2.04
RunWk	2	.	.	.	.	.
RunWk	3	0.3040	0.1955	1.36	0.92	1.99
PTDist		0.0017	0.0007	1.00	1.00	1.00
Hardiness NegSum		0.0174	0.0113	1.02	1.00	1.04
PSQIGlobal		0.0849	0.0337	1.09	1.02	1.16
SleepQual	0	.	.	.	.	.
SleepQual	1	-0.0898	0.1868	0.91	0.63	1.32
SleepQual	2	-0.5994	0.2840	0.55	0.31	0.96
SleepQual	3	0.1217	0.5295	1.13	0.40	3.19

Std Error= Standard Error
LowerCL= Lower limit of 95% Confidence Interval of Odds Ratio
UpperCL= Upper limit of 95% Confidence Interval of Odds Ratio

© 2025 Foulis SA et al. *JAMA Network Open*

eTable 5: Male-specific model, regression model details (SAS output)

Parameter	Class	Estimate	Std Error	OddsRatioEst	LowerCL	UpperCL
Intercept		-0.3922	0.0470	0.68	.	.
Age		0.0569	0.0145	1.06	1.03	1.09
Race	1	0.4587	0.1717	1.58	1.13	2.22
Race	2	-0.1258	0.2761	0.88	0.51	1.51
Race	3	0.1650	0.1347	1.18	0.91	1.54
Race	4	0.3205	0.1872	1.38	0.95	1.99
Race	5	.	.	.	.	.
BodyFat		0.0381	0.0096	1.04	1.02	1.06
BMD		-1.8473	0.4414	0.16	0.07	0.38
VitD		-0.0027	0.0061	1.00	0.99	1.01
TIBC		-0.0032	0.0014	1.00	0.99	1.00
Ferritin		-0.0017	0.0008	1.00	1.00	1.00
Tobacco	0	.	.	.	.	.
Tobacco	1	0.2470	0.1153	1.28	1.02	1.60
SF	0	.	.	.	.	.
SF	1	0.4304	0.2895	1.54	0.87	2.71
BB	0	.	.	.	.	.
BB	1	0.3407	0.1223	1.41	1.11	1.79
LoadClass	1	0.0388	0.1440	1.04	0.78	1.38
LoadClass	2	0.1770	0.2027	1.19	0.80	1.78
LoadClass	3	.	.	.	.	.
ExYear	1	0.0769	0.1554	1.08	0.80	1.46
ExYear	2	0.0371	0.1470	1.04	0.78	1.38
ExYear	3	.	.	.	.	.
ExYear	4	0.1394	0.1463	1.15	0.86	1.53
ExYear	5	0.2629	0.1651	1.30	0.94	1.80
PALevel	1	0.2110	0.2012	1.23	0.83	1.83
PALevel	2	0.2104	0.1503	1.23	0.92	1.66
PALevel	3	.	.	.	.	.
PALevel	4	0.0017	0.1422	1.00	0.76	1.32
PALevel	5	0.1211	0.1826	1.13	0.79	1.61
RunWk	1	0.1650	0.1229	1.18	0.93	1.50
RunWk	2	.	.	.	.	.
RunWk	3	-0.0342	0.1292	0.97	0.75	1.24
WeightsWk	1	0.3184	0.1257	1.37	1.07	1.76
WeightsWk	2	.	.	.	.	.
WeightsWk	3	0.0869	0.1357	1.09	0.84	1.42
GritSum		0.0235	0.0088	1.02	1.01	1.04
TotalPCS		0.0088	0.0064	1.01	1.00	1.02
PSQIGlobal		0.0362	0.0235	1.04	0.99	1.09
Duration	0	.	.	.	.	.
Duration	1	-0.2526	0.1240	0.78	0.61	0.99
Duration	2	-0.2430	0.2266	0.78	0.50	1.22
Duration	3	0.3984	0.2576	1.49	0.90	2.47

Std Error= Standard Error
LowerCL= Lower limit of 95% Confidence Interval of Odds Ratio
UpperCL= Upper limit of 95% Confidence Interval of Odds Ratio

© 2025 Foulis SA et al. *JAMA Network Open*

eReferences

1. Executive summary of the clinical guidelines on the identification, evaluation, and treatment of overweight and obesity in adults. *Arch Intern Med*. Sep 28 1998;158(17):1855-67. doi:10.1001/archinte.158.17.1855
2. Foulis SA, Hughes JM, Walker LA, et al. Body mass does not reflect the body composition changes in response to similar physical training in young women and men. *Int J Obes (Lond)*. Mar 2021;45(3):659-665. doi:10.1038/s41366-020-00730-0
3. Foulis SA, Sharp MA, Redmond JE, et al. U.S. Army Physical Demands Study: Development of the Occupational Physical Assessment Test for combat arms soldiers. *J Sci Med Sport*. Nov 2017;20 Suppl 4:S74-S78. doi:10.1016/j.jsams.2017.07.018
4. Longitudinal validation of the Occupational Physical Assessment Test (OPAT). Technical Report #T18-05 (U.S. Army Research Institute of Environmental Medicine) (2018).
5. Harman EA, Rosenstein MT, Frykman PN, Rosenstein RM, Kraemer WJ. *Estimation of power output from vertical jump and body mass*. 1998.
6. Duckworth AL, Peterson C, Matthews MD, Kelly DR. Grit: perseverance and passion for long-term goals. *J Pers Soc Psychol*. Jun 2007;92(6):1087-101. doi:10.1037/0022-3514.92.6.1087
7. Sinclair R, Oliver C. *Development and validation of a short measure of hardiness.* Walter Reed Army Institute of Research; 2004.
8. Sullivan MJ, Bishop SR, Pivik J. The pain catastrophizing scale: development and validation. *Psychological assessment*. 1995;7(4):524.
9. Osman A, Barrios FX, Gutierrez PM, Kopper BA, Merrifield T, Grittmann L. The Pain Catastrophizing Scale: further psychometric evaluation with adult samples. *J Behav Med*. Aug 2000;23(4):351-65. doi:10.1023/a:1005548801037
10. Buysse DJ, Reynolds CF, 3rd, Monk TH, Berman SR, Kupfer DJ. The Pittsburgh Sleep Quality Index: a new instrument for psychiatric practice and research. *Psychiatry Res*. May 1989;28(2):193-213. doi:10.1016/0165-1781(89)90047-4
