# Supplementary material for: Model for Musculoskeletal Injury Risk Factors Among US Army Basic Combat Trainees
Source: JAMA Netw Open. 2025 Jun 2;8(6):e2513177. doi: 10.1001/jamanetworkopen.2025.13177 (PMC12131099; doi:10.1001/jamanetworkopen.2025.13177)
Supplement: Supplement 3. — Nonauthor Collaborators. The ARIEM Reduction in Musculoskeletal Injury (ARMI) Study Team [file jamanetwopen-e2513177-s003.pdf]

Supplemental Online Content: Nonauthor Collaborators
*First name, last name, and suffix (if applicable) are required and will appear in PubMed.

*Group Name(s): The ARIEM Reduction in Musculoskeletal Injury (ARMI) Study Team							
***First Name and Middle Initial(s)**	***Last Name**	***Suffix (eg, Jr, III)**	Academic Degrees	Institution	Location (city, state/province, country)	Role or Contribution, eg, chair, principal investigator	Group (if more than 1 Group listed in the byline) and/or Subgroup (eg, Steering Committee)
Paul M.	Bartlett		B.S.	Military Performance Division, United States Army Research Institute of Environmental Medicine	Natick MA, USA	Performed data collection, Contributed to the editing of the manuscript (including data interpretation), agreed with the order of the presentation of authors, and approved the final version	
Bruce S.	Cohen		Ph.D	Military Performance Division, United States Army Research Institute of Environmental Medicine	Natick MA, USA	Performed data collection, Contributed to the editing of the manuscript (including data interpretation), agreed with the order of the presentation of authors, and approved the final version	
Peter N.	Frykman		M.S.	Military Performance Division, United States Army Research Institute of Environmental Medicine	Natick MA, USA	Performed data collection, Contributed to the editing of the manuscript (including data interpretation), agreed with the order of the presentation of authors, and approved the final version	
Alyssa V.	Geddis		B.S.	Military Performance Division, United States Army Research Institute of Environmental Medicine	Natick MA, USA	Performed data collection, Contributed to the editing of the manuscript (including data interpretation), agreed with the order of the presentation of authors, and approved the final version	
Soothesuk	Kusumpa		M.S.	Military Performance Division, United States Army Research Institute of Environmental Medicine	Natick MA, USA	Performed data collection, Conducted data management and preparation for analysis, Contributed to the editing of the manuscript (including data interpretation), agreed with the order of the presentation of authors, and approved the final version	
Caleb D.	Johnson		Ph.D.	Military Performance Division, United States Army Research Institute of Environmental Medicine	Natick MA, USA	Performed data collection, Contributed to the editing of the manuscript (including data interpretation), agreed with the order of the presentation of authors, and approved the final version	
Vy T.	Nguyen		Ph.D.	Military Performance Division, United States Army Research Institute of Environmental Medicine	Natick MA, USA	Performed data collection, Contributed to the editing of the manuscript (including data interpretation), agreed with the order of the presentation of authors, and approved the final version	
Vincent P.	Pecorelli		B.S.	Military Performance Division, United States Army Research Institute of Environmental Medicine	Natick MA, USA	Performed data collection, Conducted data management and preparation for analysis, Contributed to the editing of the manuscript (including data interpretation), agreed with the order of the presentation of authors, and approved the final version	
Marinaliz	Reynoso		M.S.	Military Performance Division, United States Army Research Institute of Environmental Medicine	Natick MA, USA	Performed data collection, Contributed to the editing of the manuscript (including data interpretation), agreed with the order of the presentation of authors, and approved the final version	

Supplemental Online Content: Nonauthor Collaborators
*First name, last name, and suffix (if applicable) are required and will appear in PubMed.

***First Name and Middle Initial(s)**	***Last Name**	***Suffix (eg, Jr, III)**	Academic Degrees	Institution	Location (city, state/province, country)	Role or Contribution, eg, chair, principal investigator	Group (if more than 1 Group listed in the byline) and/or Subgroup (eg, Steering Committee)
Melissa D.	Richardson		M.P.H.	Military Performance Division, United States Army Research Institute of Environmental Medicine	Natick MA, USA	Conducted data management and preparation for analysis, Contributed to the editing of the manuscript (including data interpretation), agreed with the order of the presentation of authors, and approved the final version	
Nathaniel I.	Smith		M.S.	Military Performance Division, United States Army Research Institute of Environmental Medicine	Natick MA, USA	Performed data collection, Contributed to the editing of the manuscript (including data interpretation), agreed with the order of the presentation of authors, and approved the final version	
David J.	Zeppetelli		B.S.	Military Performance Division, United States Army Research Institute of Environmental Medicine	Natick MA, USA	Performed data collection, Conducted data management and preparation for analysis, Contributed to the editing of the manuscript (including data interpretation), agreed with the order of the presentation of authors, and approved the final version	
Bradley C.	Nindl		Ph.D.	University of Pittsburgh Neuromuscular Research Laboratory/Warrior Human Performance Research Center, Department of Sports Medicine and Nutrition, School of Health and Rehabilitation Sciences	Pittsburgh, PA, USA	Contributed to the editing of the manuscript (including data interpretation), agreed with the order of the presentation of authors, and approved the final version	
Shawn D.	Flanagan		Ph.D.	University of Pittsburgh Neuromuscular Research Laboratory/Warrior Human Performance Research Center, Department of Sports Medicine and Nutrition, School of Health and Rehabilitation Sciences	Pittsburgh, PA, USA	Contributed to the editing of the manuscript (including data interpretation), agreed with the order of the presentation of authors, and approved the final version	
Ryan S.	Sacko		Ph.D.	Department of Health and Human Performance, The Citadel	Charleston, SC, USA	Contributed to the editing of the manuscript (including data interpretation), agreed with the order of the presentation of authors, and approved the final version	
