# Supplementary material for: Model for Musculoskeletal Injury Risk Factors Among US Army Basic Combat Trainees
Source: JAMA Netw Open. 2025 Jun 2;8(6):e2513177. doi: 10.1001/jamanetworkopen.2025.13177 (PMC12131099; doi:10.1001/jamanetworkopen.2025.13177)
Supplement: Supplement 4. — Data Sharing Statement [file jamanetwopen-e2513177-s004.pdf]

Data Sharing Statement

Foulis. Model for Musculoskeletal Injury Risk Factors Among US Army Basic Combat Trainees. *JAMA Netw Open*. Published June 02, 2025. doi:10.1001/jamanetworkopen.2025.13177

Data

Data types: Raw data and computational spreadsheet for example scenarios of traffic light musculoskeletal injury risk
How to access data: The unlocked version of the computational spreadsheet can be made available upon reasonable request by emailing kathryn.m.taylor41.civ@health.mil
When available: With publication
Data available: No

Additional Information

Explanation for why data not available: The raw data underlying this article cannot be shared publicly due to government restrictions. Data will only be provided after a formal request is made to the corresponding author and if individual permission is given for its release by the U.S. Army and Department of Defense data owners.
